# Development and external validation of a nomogram for predicting one-year survival in patients with non-traumatic subarachnoid hemorrhage

**DOI:** 10.3389/fsurg.2025.1579429

**Published:** 2025-09-12

**Authors:** Yiwei Lv, Zhongsheng Lu, Menghui He, Zihai Cheng, Qiang Zhang, Xiaoqing Jin, Pei Han

**Affiliations:** ^1^ Department of Graduate School, Qinghai University, Xining, China; ^2^ Department of Neurosurgery, Qinghai Provincial People’s Hospital, Xining, China; ^3^ Qinghai Provincial People’s Hospital, Xining, China

**Keywords:** external validation, medical information mart for intensive care, nomogram, nontraumatic subarachnoid hemorrhage, survival

## Abstract

**Background:**

Subarachnoid hemorrhage (SAH), a critical cerebrovascular emergency characterized by acute bleeding into the subarachnoid space, is associated with permanent neurological deficits, substantial mortality rates, and unfavorable clinical outcomes. Survivors frequently develop long-term complications including cognitive impairment, memory loss, and neuropsychiatric issues like depression, anxiety, and PTSD, significantly reducing quality of life. Despite advancements in acute-phase management, the long-term survival prognosis for non-traumatic SAH patients remains poorly characterized in current clinical research. Identifying reliable prognostic biomarkers and developing validated predictive models are crucial for enabling risk-stratified care and personalized treatments, improving evidence-based clinical practice.

**Method:**

This study analyzed baseline and clinical data from 825 non-traumatic SAH patients in the MIMIC-IV ICU database. Kaplan–Meier analysis and multivariate Cox regression identified independent survival risk factors, followed by nomogram model construction. The model's performance was evaluated using C-index, ROC curve (AUC), calibration curve, and DCA to assess discrimination, calibration, and clinical utility. External validation was performed using 290 non-traumatic SAH patients from Qinghai Provincial People's Hospital.

**Result:**

Multivariate Cox regression identified 11 independent risk factors for non-traumatic SAH survival: hospital stay length, age, respiratory rate, red blood cell count, platelets, potassium, sodium, anion gap, urea nitrogen, blood glucose, and sepsis. A nomogram model based on these factors showed strong discrimination, stratifying patients into risk categories. In the training cohort, the model achieved an AUC of 0.844 (95% CI: 0.815–0.872) and a C-index of 0.827 (95% CI: 0.803–0.851). In the external validation set, the model exhibited acceptable discriminatory performance, with an AUC of 0.807 (95% CI: 0.758–0.856) and a C-index of 0.851 (95% CI: 0.825–0.875).

**Conclusion:**

In this study, the survival prognosis of patients with non-traumatic subarachnoid hemorrhage (SAH) was found to be associated with eleven factors: length of hospital stay, patient age, respiratory rate, red blood cell count, platelet count, potassium levels, sodium levels, anion gap, urea nitrogen, blood glucose levels, and the presence of sepsis. The nomogram model we developed demonstrates superior predictive accuracy and can serve as a valuable tool for clinicians in rapidly identifying high-risk patients, facilitating personalized risk assessment, and guiding targeted medical interventions.

## Introduction

Subarachnoid hemorrhage (SAH) is a cerebrovascular disorder characterized by the acute onset of neurological dysfunction caused by intracranial bleeding into the subarachnoid space, which can arise from various etiologies. SAH represents one of the most severe forms of stroke and is often associated with a poor prognosis. Common complications include hydrocephalus, cerebral edema, delayed cerebral ischemia, epilepsy, and electrolyte metabolic disturbances (1). Approximately 85% of spontaneous subarachnoid hemorrhages are caused by aneurysm rupture (2). Approximately 10% of cases are classified as non-aneurysmal perimesencephalic hemorrhage, while the remaining 5% are attributed to other causes. The global incidence of aneurysmal subarachnoid hemorrhage (aSAH) is estimated at 6.1 cases per 100,000 individuals annually (3). Notably, the incidence rate in women is 1.3 times higher than that observed in men (2, 4). There are significant geographical variations in the incidence rate, with the highest rates currently observed in Japan (22.7 per 100,000) and Finland (19.7 per 100,000) (5). Over the past three decades, the global incidence rate has decreased due to reduced smoking prevalence, advancements in hypertension management, and enhanced treatment protocols for unruptured aneurysms (3). Advancements in medical technology have enabled the development of more effective strategies for rapid diagnosis and early intervention, consequently leading to a reduction in mortality rates (6). Nevertheless, 15% of patients succumb to a ruptured aneurysm, and the 30-day mortality rate for patients experiencing aneurysm rupture reaches as high as 45% (3, 7). According to research conducted by Wen-Jun Tu et al. using the Big Data Observational Platform for Stroke in China (BOSC), the one-year post-discharge mortality rate for Chinese patients with subarachnoid hemorrhage (SAH) is 14%. Notably, this statistic excludes both in-hospital deaths and patients who failed to reach medical facilities in a timely manner (8). Given that the primary patient group predominantly falls within the age range of 50–55 years, this condition significantly impairs their quality of life and leads to a substantial loss of disability-free life years (9). In China, the growing aging population and the increasing prevalence of risk factors have resulted in a rising burden of stroke, particularly pronounced in rural areas. This issue is further compounded by insufficient primary prevention measures, socio-economic challenges, and widespread risk factors such as hypertension and smoking (10). Subarachnoid hemorrhage remains a significant public health concern. Hypertension, smoking, and advanced age have been identified as established significant risk factors that adversely affect survival rates (2). Accurately predicting patient survival, however, remains a complex and multifaceted challenge. To tackle this issue, developing a nomogram model to estimate survival probabilities represents a promising approach. Therefore, there is an urgent need for a rapid and efficient evaluation method to accurately determine patient survival rates and to optimize treatment strategies. In clinical research, nomogram models serve as both visual aids and statistical prediction tools, effectively forecasting the likelihood of specific clinical events. To date, however, there has been a paucity of studies dedicated to constructing nomogram models for predicting survival rates in patients with non-traumatic subarachnoid hemorrhage (11). A considerable number of predictive models have been developed and implemented in clinical settings. However, most of these studies primarily rely on traditional logistic regression analysis, with limited exploration of Cox regression, a method specifically designed for handling time-to-event data, including the FRESH score (12) and the SAHIT scoring system (13). Few studies have delved into prediction models based on survival analysis using Cox regression for forecasting long-term prognosis. This retrospective study leverages the MIMIC-IV database to investigate non-traumatic critically ill patients with subarachnoid hemorrhage (SAH). The primary objective is to develop and validate a nomogram-based prediction model that identifies key risk factors influencing patient outcomes and accurately predicts survival rates. The aim is to equip clinicians with an intuitive tool for predicting patient survival status, providing valuable insights for clinical prevention and treatment, and ultimately facilitating personalized risk assessment.

## Materials and methods

### Data sources

This retrospective study comprised two distinct cohorts: an internal cohort (*n* = 825) and an external cohort (*n* = 290). The internal cohort was derived from the MIMIC-IV database (version 2.2), an extensive, publicly available critical care database documenting 299,712 ICU or emergency department admissions at Beth Israel Deaconess Medical Center between 2008 and 2019 (https://mimic.physionet.org/) (14, 15). The external cohort consisted of 290 patients diagnosed with non-traumatic subarachnoid hemorrhage who were admitted to the ICU at Qinghai Provincial People's Hospital from January 1, 2013, to December 31, 2023. Data regarding patient demographics and treatment details were extracted from the hospital's medical records. Blood tests were performed within 24 h of ICU admission.

Access to the MIMIC-IV (v2.2) database was authorized by the Institutional Review Boards (IRBs) of MIT and Beth Israel Deaconess Medical Center (BIDMC). The selection of this database for our study is justified for several reasons. First, all patient data in MIMIC-IV undergo rigorous de-identification, wherein patient identities are replaced with randomized identifiers. This anonymization process is integral to the MIMIC-IV design, ensuring support for diverse research and educational activities while preserving patient confidentiality and facilitating clinical research. Consequently, the requirement for informed consent was waived by the BIDMC IRB (12, 13). This study adhered to the ethical guidelines of the Declaration of Helsinki and followed the principles of the Strengthening the Reporting of Observational Studies in Epidemiology (STROBE) statement. Furthermore, author Yiwei Lv successfully completed the Collaborative Institutional Training Initiative (CITI) program, passing the “Data or Specimens Only Research” examination (ID: 63743885), thereby obtaining authorized database access. The study protocol was reviewed and approved by the Clinical Research Ethics Committee of Qinghai Provincial People's Hospital.

### Data extraction and definition

The criteria for data extraction in this study were as follows: all laboratory tests and vital signs were extracted as the first measurement obtained within 24 h of ICU admission. The counting of acute clinical event covered the entire hospitalization period. The neurological status was assessed using the Glasgow Coma Scale (GCS) score recorded at admission.

### Inclusion and exclusion criteria

**Figure 1:**
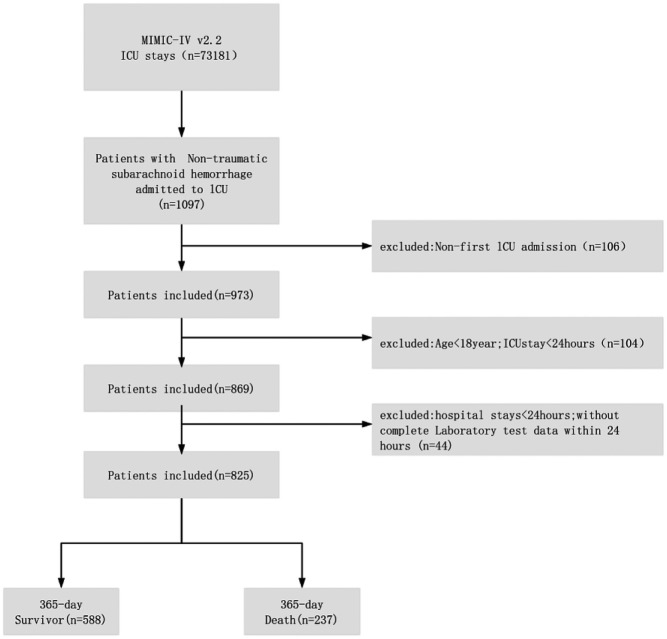
Admittance flow chart.

As depicted in Figure 1, the inclusion criteria for the internal cohort were: (1) confirmed diagnosis of non-traumatic subarachnoid hemorrhage (ICD-9: 430; ICD-10: I60, I60.0–I60.12, I60.00–I60.02, I60.20–I60.22, I60.30–I60.32, I60.50–I60.52); (2) initial ICU admission with ≥24 h stay; (3) age ≥18 years; and (4) initial hospitalization ≥24 h. Exclusion criteria were: (1) hospital stay <24 h; (2) ICU stay <24 h; (3) ICU readmission; and (4) >5% missing data. Initial screening using these diagnostic codes identified 1,176 patients from the MIMIC-IV database. Ultimately, 825 patients were enrolled in the internal cohort, while 351 were excluded for the following reasons: non-initial ICU admission (*n* = 203), ICU stay <24 h (*n* = 90), age <18 years (*n* = 14), and incomplete or invalid data (*n* = 44).

For the external cohort, inclusion required: (1) admission diagnosis of non-traumatic subarachnoid hemorrhage; (2) first ICU admission with ≥24-hour stay; (3) age ≥18 years; and (4) initial hospitalization ≥24 h. Exclusion criteria included: (1) hospital stay <24 h; (2) ICU readmission; (3) >5% data missingness; and (4) loss to follow-up. A total of 1,277 patients were initially identified. After screening, 290 patients were included in the external cohort, and 987 were excluded due to either no ICU admission (*n* = 928) or incomplete data/loss to follow-up (*n* = 59).

### Data preprocessing

Variables exceeding 5% missingness were excluded. For variables with missing rates <5%, multiple imputation was performed using the Multiple Imputation by Chained Equations (MICE) method (16, 17), which generated five complete datasets through iterative modeling. Final results were obtained by pooling these datasets to minimize imputation uncertainty. The imputation was conducted using the “mice” package in R software, and its quality was validated according to the diagnostic procedures recommended by van Buuren (18).

### Variable selection and model construction

We prospectively collected multidimensional clinical data comprising: Demographic parameters: age, sex, hospitalization duration, ICU length of stay;Laboratory indices: hematological parameters (red blood cell [RBC], platelet, white blood cell [WBC] counts), serum biochemical markers (creatinine, chloride, potassium, sodium, anion gap, bicarbonate, blood urea nitrogen [BUN], glucose); Physiological measurements: mean arterial pressure (MAP), systolic/diastolic blood pressure, respiratory rate, body temperature, oxygen saturation (SpO_2_);Comorbidities: hypertension, diabetes mellitus, congestive heart failure, chronic kidney disease, peripheral vascular disease, chronic pulmonary disease, cerebrovascular disease; Therapeutic interventions: nimodipine administration, levetiracetam therapy, vasopressor use, mechanical ventilation, renal replacement therapy; Acute clinical events: sepsis, acute kidney injury; Neurosurgical procedures: endovascular coiling, surgical clipping; Neurological status: Glasgow Coma Scale (GCS) scores. The validation cohort included 11 clinically validated prognostic variables: hospitalization duration, age, respiratory rate, RBC count, platelet count, serum potassium, serum sodium, anion gap, BUN, blood glucose, and sepsis occurrence.

This study has the following methodological limitations: (a) Two widely used tools for assessing subarachnoid hemorrhage (SAH) severity—the Hunt-Hess grade and modified Fisher scale—were not included due to limited data availability; (b) The Glasgow Coma Scale (GCS) score was dichotomized, which may not fully capture the non-linear relationship between consciousness levels and prognosis. Although these deficiencies in severity assessment were partially mitigated by multidimensional laboratory and physiological parameters, these limitations could affect the model's comprehensive evaluation of the initial clinical status.

### Statistic analysis

Survival analyses were conducted using Kaplan–Meier estimates with log-rank tests for group comparisons. Continuous variables are presented as mean ± standard deviation (SD) and compared using Student's *t*-tests or Wilcoxon rank-sum tests, while categorical variables were analyzed with chi-square (*χ*^2^) tests. A forest plot was generated to visualize associations between risk factors and survival outcomes. Variable selection employed a dual approach: (1) univariate Cox regression (significance threshold: *P* < 0.05) and (2) LASSO regression with 10-fold cross-validation (optimal *λ* selected by minimum mean squared error). Variables identified by both methods were entered into multivariate Cox proportional hazards regression. Stepwise backward selection was then applied to derive the final variable set with the lowest Akaike Information Criterion (AIC). The nomogram model was constructed based on Cox regression results. Its discriminatory ability was assessed using the receiver operating characteristic (ROC) curve, with the area under the curve (AUC) as the primary metric. The nomogram demonstrated superior performance compared to alternatives, showing higher AUC (0.844 vs. 0.819) and lower AIC (2,743.23 vs. 2,916.62). Calibration curves were generated with 1,000 bootstrap samples to evaluate prediction accuracy. Decision curve analysis (DCA) quantified clinical utility and net benefit. Using R software (version 4.4.2), scores were assigned to each risk factor. Total scores enabled calculation of individual survival probabilities, and a risk stratification map was created for clinical application.

The primary endpoint of this study is the survival status at 365 days. The mortality rate of patients one year after discharge. For patients who died during hospitalization, survival time was defined as the interval from admission to death. For discharged patients, survival time was calculated as the interval from admission to the last follow-up date. For cases without death within 365 days but with incomplete survival data, survival time was censored at 365 days. Optimal cutoff values for continuous variables were determined using X-tile software, and parameters were categorized into high, medium, and low groups based on these cutoffs. All statistical analyses were performed using R software (version 4.4.2), with results reported as hazard ratios (HRs) and corresponding 95% confidence intervals (CIs). All tests were two-sided, and a *p*-value below 0.05 was considered statistically significant.

## Result

### Baseline characterization

This study aimed to analyze the clinical characteristics of patients with non-traumatic subarachnoid hemorrhage (SAH) from an internal cohort. The study subjects were divided into a training set (*n* = 825) and a validation set (*n* = 290), as shown in Table 1. The intergroup difference analysis of the patients in the training set revealed that among them, 588 had a good prognosis and survived, accounting for 71.27%, and 237 died within one year, accounting for 28.73%. There were statistically significant differences in Length of stay, Intensive Care Unit length of Stay, age, red blood cell count, creatinine, chloride, platelets, potassium, sodium, anion gap, bicarbonate, blood urea nitrogen, blood glucose, heart rate, systolic blood pressure, diastolic blood pressure, mean arterial pressure, respiratory rate, temperature, heart failure, renal failure, chronic pulmonary disease, diabetes mellitus, nimodipine icu used, levetiracetam icu used, mechanical Ventilation, continuous renal replacement therapy, acute kidney injury, sepsis and vascular embolization among the groups (*P* < 0.05), while there were no statistically significant differences in white blood cell count, blood oxygen saturation, Glasgow Coma Scale, Gender, hypertension, peripheral vascular disease and vascular occlusion among the groups (*P* > 0.05).

**Table 1:** Baseline characteristics of patients with non-traumatic subarachnoid hemorrhage.

Variables	Total (*n* = 825)	0 (*n* = 588)	1 (*n* = 237)	Statistic	*P*
LOS, *n* (%)				*χ*^2^ = 77.59	<0.001
<3.96	114 (13.82)	42 (7.14)	72 (30.38)		
>15.09	289 (35.03)	216 (36.73)	73 (30.80)		
3.96–15.09	422 (51.15)	330 (56.12)	92 (38.82)		
ICU LOS, *n* (%)				*χ*^2^ = 18.56	<0.001
<2.04	131 (15.88)	79 (13.44)	52 (21.94)		
>4.88	497 (60.24)	381 (64.80)	116 (48.95)		
2.04–4.88	197 (23.88)	128 (21.77)	69 (29.11)		
Age, *n* (%)				*χ*^2^ = 75.85	<0.001
<56.69	334 (40.48)	275 (46.77)	59 (24.89)		
>77.58	149 (18.06)	65 (11.05)	84 (35.44)		
56.69–77.58	342 (41.45)	248 (42.18)	94 (39.66)		
RBC, *n* (%)				*χ*^2^ = 55.15	<0.001
<3.37	124 (15.03)	54 (9.18)	70 (29.54)		
>3.96	447 (54.18)	344 (58.50)	103 (43.46)		
3.37–3.96	254 (30.79)	190 (32.31)	64 (27.00)		
Cr, *n* (%)				*χ*^2^ = 66.40	<0.001
<0.60	85 (10.30)	66 (11.22)	19 (8.02)		
>1.10	104 (12.61)	39 (6.63)	65 (27.43)		
0.60–1.10	636 (77.09)	483 (82.14)	153 (64.56)		
Cl, *n* (%)				*χ*^2^ = 8.15	0.017
<103.00	258 (31.27)	167 (28.40)	91 (38.40)		
>106.00	272 (32.97)	199 (33.84)	73 (30.80)		
103.00–106.00	295 (35.76)	222 (37.76)	73 (30.80)		
PLT, *n* (%)				*χ*^2^ = 37.20	<0.001
<137.00	92 (11.15)	43 (7.31)	49 (20.68)		
>186.00	549 (66.55)	422 (71.77)	127 (53.59)		
137.00–186.00	184 (22.30)	123 (20.92)	61 (25.74)		
WBC, *n* (%)				*χ*^2^ = 3.47	0.176
<8.40	177 (21.45)	133 (22.62)	44 (18.57)		
>11.10	432 (52.36)	296 (50.34)	136 (57.38)		
8.40–11.10	216 (26.18)	159 (27.04)	57 (24.05)		
K, *n* (%)				*χ*^2^ = 25.73	<0.001
<3.50	137 (16.61)	91 (15.48)	46 (19.41)		
>4.50	94 (11.39)	48 (8.16)	46 (19.41)		
3.50–4.50	594 (72.00)	449 (76.36)	145 (61.18)		
Na, *n* (%)				*χ*^2^ = 12.77	0.002
<137.00	167 (20.24)	110 (18.71)	57 (24.05)		
>141.00	202 (24.48)	130 (22.11)	72 (30.38)		
137.00–141.00	456 (55.27)	348 (59.18)	108 (45.57)		
AG, *n* (%)				*χ*^2^ = 27.47	<0.001
<12.00	106 (12.85)	81 (13.78)	25 (10.55)		
>18.00	85 (10.30)	40 (6.80)	45 (18.99)		
12.00–18.00	634 (76.85)	467 (79.42)	167 (70.46)		
HCO_3_, *n* (%)				*χ*^2^ = 24.27	<0.001
<20.00	101 (12.24)	51 (8.67)	50 (21.10)		
>22.00	457 (55.39)	339 (57.65)	118 (49.79)		
20.00–22.00	267 (32.36)	198 (33.67)	69 (29.11)		
BUN *n* (%)				*χ*^2^ = 98.47	<0.001
<17.00	559 (67.76)	454 (77.21)	105 (44.30)		
>24.00	88 (10.67)	31 (5.27)	57 (24.05)		
17.00–24.00	178 (21.58)	103 (17.52)	75 (31.65)		
Glu, *n* (%)				*χ*^2^ = 27.39	<0.001
<118.00	287 (34.79)	223 (37.93)	64 (27.00)		
>218.00	88 (10.67)	43 (7.31)	45 (18.99)		
118.00–218.00	450 (54.55)	322 (54.76)	128 (54.01)		
HR, *n* (%)				*χ*^2^ = 10.88	0.004
<76.00	360 (43.64)	273 (46.43)	87 (36.71)		
>98.00	121 (14.67)	73 (12.41)	48 (20.25)		
76.00–98.00	344 (41.70)	242 (41.16)	102 (43.04)		
SBP, *n* (%)				*χ*^2^ = 7.32	0.026
<118.00	203 (24.61)	137 (23.30)	66 (27.85)		
>129.00	456 (55.27)	319 (54.25)	137 (57.81)		
118.00–129.00	166 (20.12)	132 (22.45)	34 (14.35)		
DBP, *n* (%)				*χ*^2^ = 7.14	0.028
<57.00	130 (15.76)	80 (13.61)	50 (21.10)		
>65.00	520 (63.03)	380 (64.63)	140 (59.07)		
57.00–65.00	175 (21.21)	128 (21.77)	47 (19.83)		
MAP, *n* (%)				*χ*^2^ = 11.37	0.003
<70.50	98 (11.88)	58 (9.86)	40 (16.88)		
>80.00	559 (67.76)	398 (67.69)	161 (67.93)		
70.50–80.00	168 (20.36)	132 (22.45)	36 (15.19)		
RR, *n* (%)				*χ*^2^ = 25.59	<0.001
<15.50	258 (31.27)	205 (34.86)	53 (22.36)		
>22.00	120 (14.55)	65 (11.05)	55 (23.21)		
15.50–22.00	447 (54.18)	318 (54.08)	129 (54.43)		
T, *n* (%)				*χ*^2^ = 16.18	<0.001
<36.61	251 (30.42)	158 (26.87)	93 (39.24)		
>37.44	92 (11.15)	61 (10.37)	31 (13.08)		
36.61–37.44	482 (58.42)	369 (62.76)	113 (47.68)		
SpO_2_, *n* (%)				*χ*^2^ = 5.14	0.077
<97.00	213 (25.82)	157 (26.70)	56 (23.63)		
>99.00	309 (37.45)	206 (35.03)	103 (43.46)		
97.00–99.00	303 (36.73)	225 (38.27)	78 (32.91)		
GCS, *n* (%)				*χ*^2^ = 1.49	0.222
>13.00	715 (86.67)	515 (87.59)	200 (84.39)		
3.00–13.00	110 (13.33)	73 (12.41)	37 (15.61)		
Gender, *n* (%)				*χ*^2^ = 0.96	0.328
F	464 (56.24)	227 (54.57)	237 (57.95)		
M	361 (43.76)	189 (45.43)	172 (42.05)		
HBP, *n* (%)				*χ*^2^ = 0.72	0.397
0	416 (50.42)	302 (51.36)	114 (48.10)		
1	409 (49.58)	286 (48.64)	123 (51.90)		
HF, *n* (%)				*χ*^2^ = 8.84	0.003
0	763 (92.48)	554 (94.22)	209 (88.19)		
1	62 (7.52)	34 (5.78)	28 (11.81)		
RF, *n* (%)				*χ*^2^ = 57.93	<0.001
0	732 (88.73)	553 (94.05)	179 (75.53)		
1	93 (11.27)	35 (5.95)	58 (24.47)		
PVD, *n* (%)				*χ*^2^ = 1.24	0.265
0	765 (92.73)	549 (93.37)	216 (91.14)		
1	60 (7.27)	39 (6.63)	21 (8.86)		
CPD, *n* (%)				*χ*^2^ = 7.87	0.005
0	709 (85.94)	518 (88.10)	191 (80.59)		
1	116 (14.06)	70 (11.90)	46 (19.41)		
DM, *n* (%)				*χ*^2^ = 4.90	0.027
0	710 (86.06)	516 (87.76)	194 (81.86)		
1	115 (13.94)	72 (12.24)	43 (18.14)		
NM, *n* (%)				*χ*^2^ = 38.48	<.0001
0	362 (43.88)	218 (37.07)	144 (60.76)		
1	463 (56.12)	370 (62.93)	93 (39.24)		
LEV, *n* (%)				*χ*^2^ = 4.18	0.041
0	334 (40.48)	225 (38.27)	109 (45.99)		
1	491 (59.52)	363 (61.73)	128 (54.01)		
MV, *n* (%)				*χ*^2^ = 7.44	0.006
0	199 (24.12)	157 (26.70)	42 (17.72)		
1	626 (75.88)	431 (73.30)	195 (82.28)		
CRRT, *n* (%)				*χ*^2^ = 24.68	<0.001
0	809 (98.06)	586 (99.66)	223 (94.09)		
1	16 (1.94)	2 (0.34)	14 (5.91)		
AKI, *n* (%)				*χ*^2^ = 9.12	0.003
0	232 (28.12)	183 (31.12)	49 (20.68)		
1	593 (71.88)	405 (68.88)	188 (79.32)		
Sepsis, *n* (%)				*χ*^2^ = 43.37	<0.001
0	417 (50.55)	340 (57.82)	77 (32.49)		
1	408 (49.45)	248 (42.18)	160 (67.51)		
VE, *n* (%)				*χ*^2^ = 9.91	0.002
0	701 (84.97)	485 (82.48)	216 (91.14)		
1	124 (15.03)	103 (17.52)	21 (8.86)		
VO, *n* (%)				*χ*^2^ = 2.40	0.122
0	790 (95.76)	559 (95.07)	231 (97.47)		
1	35 (4.24)	29 (4.93)	6 (2.53)		

Group comparisons were performed using *χ*^2^ test (categorical variables).

LOS, length of stay; ICU LOS, intensive care unit length of stay; Age, age; Gender, male or female; RBC, red blood cell count; Cr, creatinine; Cl, chloride; PLT, platelet; WBC, white blood cell count; K, potassium; Na, sodium; AG, anion gap; HCO_3_, bicarbonate; BUN, blood urea nitrogen; Glu, blood glucose; HR, heart rate; SBP, systolic blood pressure; DBP, diastolic blood pressure; MAP, mean arterial pressure; RR, respiratory rate; T, temperature; SpO_2_, blood oxygen saturation; GCS, glasgow coma scale; HBP, hypertension; HF, heart failure; RF, renal failure; PVD, peripheral vascular disease; CPD, chronic pulmonary disease; DM, diabetes mellitus; CVD, cerebrovascular disease; NM, nimodipine ICU used; LEV, levetiracetam Icu used; MV, mechanical ventilation; CRRT, continuous renal replacement therapy; AKI, acute kidney injury; Sepsis, sepsis; VE, vascular embolization; VO, vascular occlusion.

### Screening factors

This study employed univariate analysis, as detailed in Table 2, to investigate the association between various clinical variables and the survival prognosis of patients with non-traumatic subarachnoid hemorrhage (SAH). Using Cox univariate analysis, we identified 30 significant variables out of 38 factors, all with *p*-values less than 0.05. The results indicated that several factors were associated with a reduced risk of poor prognosis, including shorter hospital stay (≤15.09 days), longer ICU stay (>4.88 days), higher red blood cell count (≥3.37 × 10^12^/L), higher chloride concentration (≥103.00 mmol/L), higher platelet count (≥137.00 × 10^9^/L), optimal potassium levels (3.5–4.5 mmol/L), optimal sodium levels (137–141 mmol/L), higher bicarbonate levels (≥20 mmol/L), systolic blood pressure within the normal range (118–129 mmHg), diastolic blood pressure (≥57 mmHg), mean arterial pressure (≥70.5 mmHg), body temperature within the normal range (36.61–37.44°C), and the use of nimodipine, levetiracetam, and vascular embolization therapy, all of which had hazard ratios (HR) < 1. Conversely, factors such as older age (≥56.69 years), elevated creatinine levels (>1.10 mg/dl), high potassium levels (>4.5 mmol/L), increased anion gap (>18.00 mmol/L), elevated urea nitrogen (≥17 mg/dl), high blood glucose (≥118.00 mg/dl), tachycardia (>98 beats/min), tachypnea (≥15.5 breaths/min), comorbidities like heart failure, renal failure, chronic obstructive pulmonary disease, diabetes, acute kidney injury, sepsis, as well as the use of epinephrine and renal replacement therapy, were associated with an increased risk of poor prognosis (HR > 1). Other indicators did not significantly impact patient survival prognosis.

**Table 2:** Single factor Cox regression model for predicting survival and prognosis of non traumatic patients.

Variables	*β*	S.E	*Z*	*P*	HR (95%CI)
LOS					
<3.96					1.000 (Reference)
>15.09	−1.651	0.167	−9.874	<.001	0.192 (0.138–0.266)
3.96–15.09	−1.746	0.158	−11.032	<.001	0.174 (0.128–0.238)
ICU LOS					
<2.04					1.000 (Reference)
>4.88	−0.760	0.167	−4.551	<.001	0.468 (0.337–0.649)
2.04–4.88	−0.181	0.184	−0.985	0.325	0.835 (0.582–1.196)
Age					
<56.69					1.000 (Reference)
>77.58	1.357	0.170	7.967	<.001	3.883 (2.781–5.422)
56.69–77.58	0.486	0.166	2.922	0.003	1.625 (1.173–2.250)
RBC					
<3.37					1.000 (Reference)
>3.96	−1.078	0.155	−6.940	<.001	0.340 (0.251–0.461)
3.37–3.96	−1.004	0.173	−5.797	<.001	0.366 (0.261–0.514)
Cr					
<0.60					1.000 (Reference)
>1.10	1.391	0.261	5.325	<.001	4.017 (2.408–6.703)
0.60–1.10	0.098	0.243	0.404	0.686	1.103 (0.685–1.777)
Cl					
<103.00					1.000 (Reference)
>106.00	−0.313	0.157	−1.989	0.047	0.732 (0.538–0.995)
103.00–106.00	−0.408	0.157	−2.598	0.009	0.665 (0.489–0.905)
PLT					
<137.00					1.000 (Reference)
>186.00	−1.016	0.168	−6.032	<.001	0.362 (0.260–0.504)
137.00–186.00	−0.602	0.192	−3.135	0.002	0.548 (0.376–0.798)
WBC					
<8.40					1.000 (Reference)
>11.10	0.323	0.173	1.864	0.062	1.382 (0.983–1.941)
8.40–11.10	0.079	0.201	0.395	0.693	1.083 (0.731–1.604)
K					
<3.50					1.000 (Reference)
>4.50	0.511	0.209	2.451	0.014	1.667 (1.108–2.509)
3.50–4.50	−0.392	0.169	−2.314	0.021	0.676 (0.485–0.942)
Na					
<137.00					1.000 (Reference)
>141.00	0.063	0.177	0.354	0.723	1.065 (0.752–1.507)
137.00–141.00	−0.426	0.164	−2.601	0.009	0.653 (0.474–0.900)
AG					
<12.00					1.000 (Reference)
>18.00	1.100	0.250	4.408	<.001	3.005 (1.842–4.900)
12.00–18.00	0.130	0.214	0.606	0.545	1.139 (0.748–1.734)
HCO_3_					
<20.00					1.000 (Reference)
>22.00	−0.896	0.169	−5.304	<.001	0.408 (0.293–0.568)
20.00–22.00	−0.868	0.186	−4.670	<.001	0.420 (0.292–0.604)
BUN					
<17.00					1.000 (Reference)
>24.00	1.603	0.165	9.697	<.001	4.968 (3.593–6.869)
17.00–24.00	0.967	0.151	6.390	<.001	2.630 (1.955–3.538)
Glu					
<118.00					1.000 (Reference)
>218.00	1.123	0.195	5.766	<.001	3.074 (2.099–4.503)
118.00–218.00	0.312	0.153	2.038	0.042	1.366 (1.012–1.844)
HR					
<76.00					1.000 (Reference)
>98.00	0.587	0.180	3.263	0.001	1.798 (1.264–2.558)
76.00–98.00	0.218	0.146	1.493	0.135	1.243 (0.934–1.655)
SBP					
<118.00					1.000 (Reference)
>129.00	−0.120	0.150	−0.802	0.423	0.887 (0.661–1.190)
118.00–129.00	−0.551	0.211	−2.610	0.009	0.576 (0.381–0.872)
DBP					
<57.00					1.000 (Reference)
>65.00	−0.432	0.165	−2.620	0.009	0.649 (0.470–0.897)
57.00–65.00	−0.432	0.203	−2.125	0.034	0.649 (0.436–0.967)
MAP					
<70.50					1.000 (Reference)
>80.00	−0.457	0.177	−2.586	0.010	0.633 (0.448–0.895)
70.50–80.00	−0.794	0.230	−3.454	<.001	0.452 (0.288–0.709)
RR					
<15.50					1.000 (Reference)
>22.00	0.984	0.193	5.110	<.001	2.676 (1.835–3.904)
15.50–22.00	0.388	0.163	2.377	0.017	1.474 (1.070–2.029)
T					
<36.61					1.000 (Reference)
>37.44	−0.140	0.207	−0.677	0.498	0.869 (0.579–1.305)
36.61–37.44	−0.577	0.140	−4.122	<.001	0.561 (0.427–0.739)
SpO_2_					
<97.00					1.000 (Reference)
>99.00	0.301	0.166	1.812	0.070	1.351 (0.976–1.871)
97.00–99.00	−0.015	0.175	−0.086	0.932	0.985 (0.699–1.389)
GCS					
>13.00					1.000 (Reference)
3.00–13.00	0.191	0.179	1.067	0.286	1.211 (0.852–1.719)
Gender					
F					1.00 (Reference)
M	0.12	0.13	0.9	0.366	1.162 (0.871–1.451)
HBP					
0					1.000 (Reference)
1	0.095	0.130	0.733	0.464	1.100 (0.853–1.419)
HF					
0					1.000 (Reference)
1	0.542	0.201	2.692	0.007	1.719 (1.159–2.551)
RF					
0					1.000 (Reference)
1	1.168	0.152	7.704	<.001	3.214 (2.388–4.326)
PVD					
0					1.000 (Reference)
1	0.218	0.229	0.953	0.341	1.243 (0.794–1.946)
CPD					
0					1.000 (Reference)
1	0.414	0.164	2.519	0.012	1.513 (1.096–2.087)
DM					
0					1.000 (Reference)
1	0.340	0.169	2.014	0.044	1.404 (1.009–1.954)
NM					
0					1.000 (Reference)
1	−0.811	0.133	−6.089	<.001	0.445 (0.342–0.577)
LEV					
0					1.000 (Reference)
1	−0.294	0.130	−2.253	0.024	0.746 (0.577–0.962)
CRRT					
0					1.000 (Reference)
1	1.609	0.277	5.812	<.001	4.999 (2.905–8.602)
MV					
0					1.000 (Reference)
1	0.426	0.170	2.504	0.012	1.531 (1.097–2.137)
AKI					
0					1.000 (Reference)
1	0.446	0.160	2.782	0.005	1.563 (1.141–2.140)
Sepsis					
0					1.000 (Reference)
1	0.858	0.139	6.180	<.001	2.358 (1.796–3.096)
VE					
0					1.000 (Reference)
1	−0.699	0.229	−3.059	0.002	0.497 (0.317–0.778)
VO					
0					1.000 (Reference)
1	−0.622	0.414	−1.503	0.133	0.537 (0.239–1.208)

LOS, length of stay; ICU LOS, intensive care unit length of stay; Age, age; Gender, male or female; RBC, red blood cell count; Cr, creatinine; Cl, chloride; PLT, platelet; WBC, white blood cell count; K, potassium; Na, sodium; AG, anion gap; HCO_3_, bicarbonate; BUN, blood urea nitrogen; Glu, blood glucose; HR, heart rate; SBP, systolic blood pressure; DBP, diastolic blood pressure; MAP, mean arterial pressure; RR, respiratory rate; T, temperature; SpO_2_, blood oxygen saturation; GCS, glasgow coma scale; HBP, hypertension; HF, heart failure; RF, renal failure; PVD, peripheral vascular disease; CPD, chronic pulmonary disease; DM, diabetes mellitus; CVD, cerebrovascular disease; NM, nimodipine ICU used; LEV, levetiracetam Icu used; MV, mechanical ventilation; CRRT, continuous renal replacement therapy; AKI, acute kidney injury; Sepsis, sepsis; VE, vascular embolization; VO, vascular occlusion.

**Figure 2:**
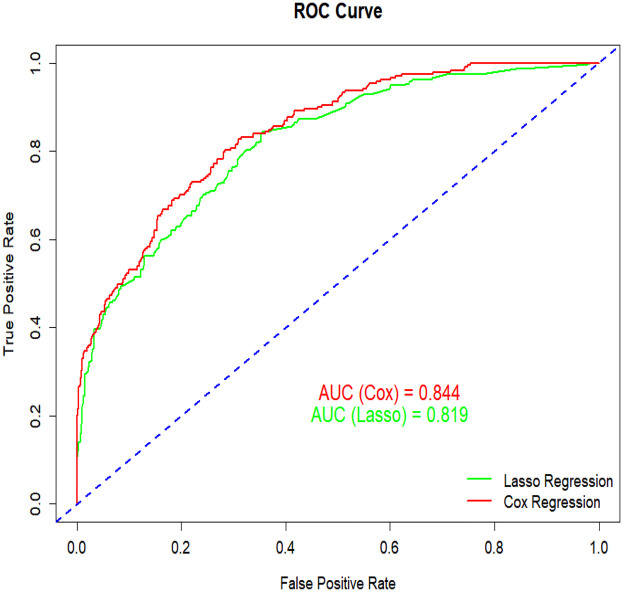
Comparison between Cox regression and lasso regression.

**Figure 3:**
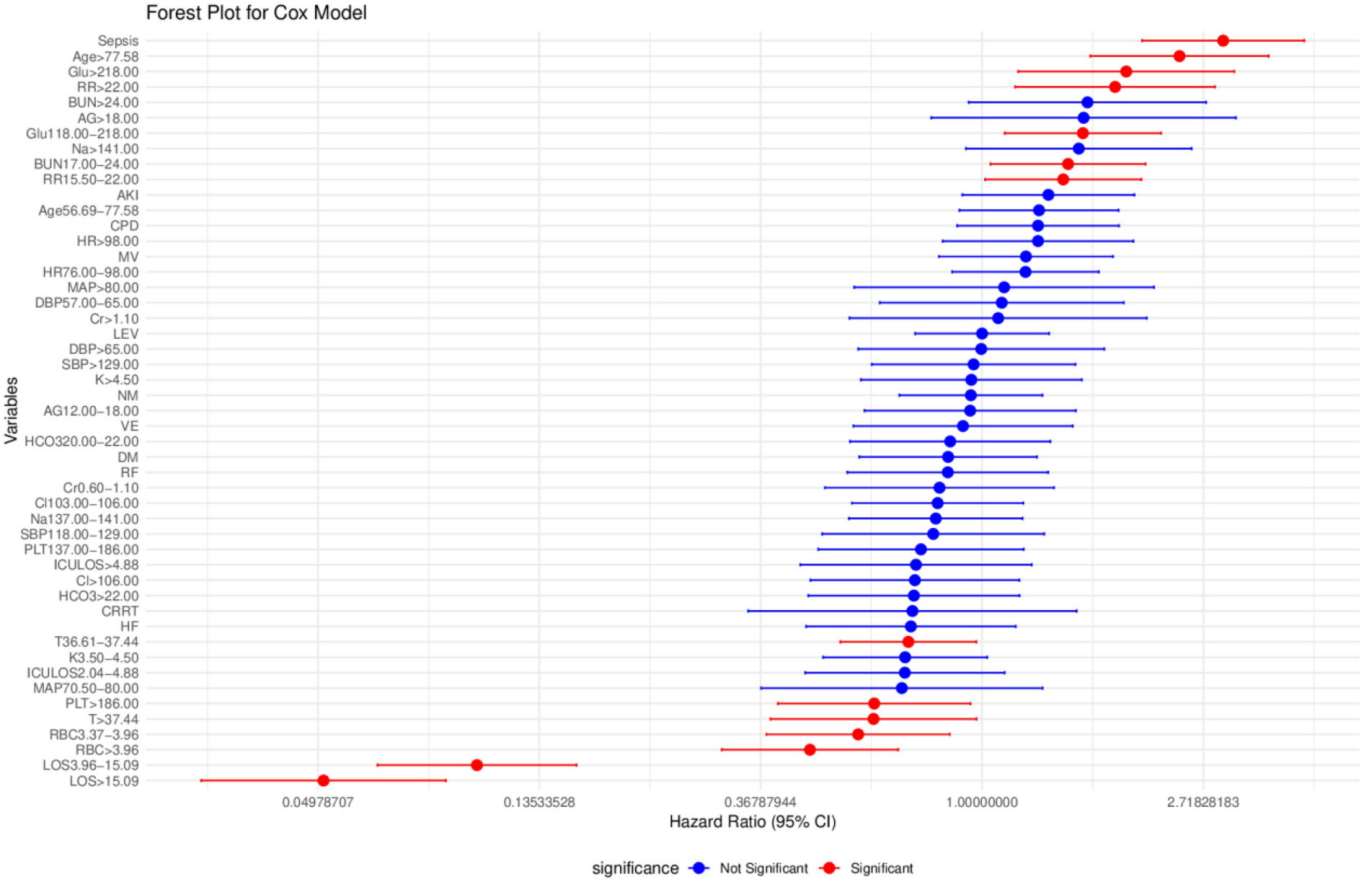
Forest plot of multivariate Cox regression analysis for survival predictors. LOS, length of stay; ICU LOS, intensive care unit length of stay; Age, age; RBC, red blood cell count; Cr, creatinine; Cl, chloride; PLT, platelet; K, potassium; Na, sodium; AG, anion gap; HCO_3_, bicarbonate; BUN, blood urea nitrogen; Glu, blood glucose; HR, heart rate; SBP, systolic blood pressure; DBP, diastolic blood pressure; MAP, mean arterial pressure; RR, respiratory rate; T, temperature; HF, heart failure; RF, renal failure; CPD, chronic pulmonary disease; DM, diabetes mellitus; CVD, cerebrovascular disease; NM, nimodipine ICU used; LEV, levetiracetam Icu used; MV, mechanical ventilation; CRRT, continuous renal replacement therapy; AKI, acute kidney injury; Sepsis, sepsis; VE, vascular embolization.

We conducted a multivariate Cox regression analysis using stepwise backward selection to identify factors influencing the independent prognosis of SAH patients, ultimately identifying 11 risk factors. As detailed in Tables 3, 4, these factors include length of hospital stay, age, respiratory rate, red blood cell count, platelet count, potassium levels, sodium levels, anion gap, urea nitrogen, blood glucose, and sepsis. To enhance the robustness of our findings, we applied Lasso regression with cross-validation, selecting the variable combination based on the *λ* value corresponding to the minimum mean square error (MSE). This process identified 8 key risk factors: length of hospital stay, body temperature, red blood cell count, urea nitrogen, renal failure, renal replacement therapy, sepsis, and nimodipine. Upon comparison, as illustrated in Figure 2, the nomogram constructed using the Cox regression model demonstrated superior performance. Therefore, this study selected the aforementioned 11 factors—length of hospital stay, age, respiratory rate, red blood cell count, platelet count, potassium levels, sodium levels, anion gap, urea nitrogen, blood glucose, and sepsis—as the foundation for constructing the nomogram model. For a more intuitive understanding of each factor's influence, please refer to the forest plot presented in Figure 3. Patient survival probabilities are presented in Table 5. Comparisons of baseline characteristics and multivariate analysis between the training and validation sets are shown in Tables 6, 7.

**Table 3:** Meaningful indicators for multi factor analysis.

Variables	coef	se	*Z*_value	*P*_value	HR	CI_lower	CI_upper
LOS ≥ 15.09	−2.9786	0.2130	−13.9842	0.0001	0.0509	0.0335	0.0772
LOS = 3.96–15.09	−2.3978	0.1865	−12.8554	0.0001	0.0909	0.0631	0.1310
Age ≥ 77.58	0.9933	0.1870	5.3124	0.0001	2.7000	1.8716	3.8951
Age = 56.69–77.58	0.3087	0.1792	1.7228	0.0849	1.3616	0.9584	1.9344
RBC ≥ 3.96	−0.7649	0.1851	−4.1335	0.0001	0.4654	0.3238	0.6688
RBC = 3.37–3.96	−0.5656	0.1972	−2.8681	0.0041	0.5680	0.3859	0.8360
PLT ≥ 186.00	−0.4630	0.1984	−2.3333	0.0196	0.6294	0.4266	0.9286
PLT = 137.00–186.00	−0.3205	0.2142	−1.4964	0.1346	0.7258	0.4770	1.1044
K ≥ 4.50	−0.2172	0.2344	−0.9269	0.3540	0.8047	0.5083	1.2740
K = 3.50–4.50	−0.4986	0.1793	−2.7806	0.0054	0.6074	0.4274	0.8632
Na ≥ 141.00	0.1753	0.1884	0.9304	0.3522	1.1916	0.8237	1.7237
Na = 137.00–141.00	−0.3722	0.1713	−2.1726	0.0298	0.6892	0.4926	0.9642
AG ≥ 18.00	0.8154	0.2825	2.8863	0.0039	2.2600	1.2991	3.9317
AG = 12.00–18.00	0.0596	0.2264	0.2632	0.7924	1.0614	0.6810	1.6544
BUN ≥ 24.00	0.4758	0.2028	2.3465	0.0190	1.6093	1.0815	2.3946
BUN = 17.00–24.00	0.4045	0.1681	2.4064	0.0161	1.4985	1.0779	2.0831
Glu ≥ 218.00	0.7173	0.2142	3.3480	0.0008	2.0488	1.3463	3.1179
Glu = 118.00–218.00	0.5938	0.1646	3.6083	0.0003	1.8109	1.3116	2.5002
RR ≥ 22.00	0.6887	0.2079	3.3120	0.0009	1.9912	1.3246	2.9930
RR = 15.50–22.00	0.4053	0.1683	2.4076	0.0161	1.4997	1.0783	2.0859
Sepsis	1.1219	0.1674	6.7016	0.0001	3.0707	2.2117	4.2632

LOS, length of stay; Age, age; RBC, red blood cell count; PLT, platelet; K, potassium; Na, sodium; AG, anion gap; BUN, blood urea nitrogen; Glu, blood glucose; RR, respiratory rate; sepsis, sepsis.

**Table 4:** The corresponding scoring system table of nomogram.

Clinical indicator	Score range	Points
RBC	<3.37	26
	3.37–3.96	7
	>3.96	0
LOS	<3.96	100
	3.96–15.09	19
	>15.09	0
PLT	<137.00	16
	137.00–186.00	5
	>186.00	0
Age	<56.69	0
	56.69–77.58	10
	>77.58	33
K	<3.50	17
	3.50–4.50	0
	>4.50	9
AG	<12.00	0
	12.00–18.00	2
	>18.00	27
Na	<137.00	12
	137.00–141.00	0
	>141.00	18
BUN	<17.00	0
	17.00–24.00	14
	>24.00	16
Glu	<118.00	0
	118.00–218.00	20
	>218.00	24
RR	<15.50	0
	15.50–22.00	14
	>22.00	23
Sepsis	0	0
	1	38

RBC, red blood cell count; LOS, length of stay; PLT, platelet; Age, age; K, potassium; AG, anion gap; BUN, blood urea nitrogen; Na, sodium; Glu, blood glucose; RR, respiratory rate; Sepsis, sepsis.

**Table 5:** The probability of prognosis corresponding to the scoring system.

Total_points	Survival_probability
0	0.99
55	0.95
79	0.9
104	0.8
120	0.7
132	0.6
142	0.5
151	0.4
161	0.3
170	0.2
182	0.1

**Table 6:** Statistical characteristics of the training set and the validation set.

Statistical characteristics of the training set							
	Variables	Total (*n* = 825)	0 (*n* = 588)	1 (*n* = 237)	*p*	Test	SMD
Gender (%)	F	464 (56.2)	337 (57.3)	127 (53.6)	0.369		0.075
	M	361 (43.8)	251 (42.7)	110 (46.4)			
LOS [mean (SD]		14.66 ± 12.40	15.22 ± 11.28	13.29 ± 14.76	0.043		0.147
ICU LOS, [mean (SD]		9.15 ± 8.30	9.55 ± 8.35	8.16 ± 8.13	0.029		0.169
Age [mean (SD]		61.49 ± 15.07	58.75 ± 14.01	68.30 ± 15.48	<0.001		0.647
Rbc [mean (SD]		4.01 ± 0.67	4.09 ± 0.58	3.83 ± 0.83	<0.001		0.364
Cr [mean (SD]		0.93 ± 0.86	0.83 ± 0.77	1.18 ± 1.00	<0.001		0.391
Cl [mean (SD]		104.52 ± 4.88	104.81 ± 4.32	103.78 ± 6.01	0.006		0.197
PLT [mean (SD]		221.97 ± 87.07	228.98 ± 81.29	204.57 ± 98.02	<0.001		0.271
Wbc [mean (SD]		12.26 ± 5.44	11.71 ± 4.61	13.63 ± 6.92	<0.001		0.326
K [mean (SD]		3.95 ± 0.64	3.93 ± 0.54	4.02 ± 0.84	0.064		0.129
Na [mean (SD]		139.20 ± 3.99	139.24 ± 3.43	139.08 ± 5.14	0.605		0.036
AG [mean (SD]		14.84 ± 3.37	14.40 ± 2.94	15.92 ± 4.05	<0.001		0.43
HCO_3_ [mean (SD]		22.87 ± 3.37	23.09 ± 2.95	22.32 ± 4.19	0.003		0.212
BUN [mean (SD]		16.37 ± 11.92	13.98 ± 7.40	22.31 ± 17.62	<0.001		0.616
Glu [mean (SD]		146.30 ± 61.17	139.40 ± 53.68	163.43 ± 74.07	<0.001		0.372
Heart.Rate [mean (SD]		81.05 ± 17.57	79.61 ± 16.59	84.63 ± 19.38	<0.001		0.278
Sbp [mean (SD]		132.79 ± 22.74	132.97 ± 21.69	132.34 ± 25.21	0.718		0.027
Dbp [mean (SD]		71.66 ± 16.18	71.85 ± 15.43	71.19 ± 17.93	0.598		0.039
Mbp [mean (SD]		88.74 ± 17.86	88.81 ± 16.59	88.56 ± 20.72	0.851		0.014
RR [mean (SD]		17.92 ± 5.01	17.34 ± 4.54	19.35 ± 5.79	<0.001		0.387
T [mean (SD]		36.80 ± 0.72	36.82 ± 0.64	36.75 ± 0.88	0.207		0.091
SpO_2_ [mean (SD]		97.68 ± 2.97	97.61 ± 2.87	97.85 ± 3.20	0.294		0.079
HBP (%)	No	416 (50.4)	302 (51.4)	114 (48.1)	0.441		0.065
	Yes	409 (49.6)	286 (48.6)	123 (51.9)			
HF (%)	No	763 (92.5)	554 (94.2)	209 (88.2)	0.005		0.214
	Yes	62 (7.5)	34 (5.8)	28 (11.8)			
RF (%)	No	732 (88.7)	553 (94.0)	179 (75.5)	<0.001		0.534
	Yes	93 (11.3)	35 (6.0)	58 (24.5)			
PVD (%)	No	765 (92.7)	549 (93.4)	216 (91.1)	0.334		0.083
	Yes	60 (7.3)	39 (6.6)	21 (8.9)			
CPD (%)	No	709 (85.9)	518 (88.1)	191 (80.6)	0.007		0.208
	Yes	116 (14.1)	70 (11.9)	46 (19.4)			
DM (%)	No	710 (86.1)	516 (87.8)	194 (81.9)	0.036		0.165
	Yes	115 (13.9)	72 (12.2)	43 (18.1)			
NM (%)	No	362 (43.9)	218 (37.1)	144 (60.8)	<0.001		0.488
	Yes	463 (56.1)	370 (62.9)	93 (39.2)			
LEV (%)	No	334 (40.5)	225 (38.3)	109 (46.0)	0.049		0.157
	Yes	491 (59.5)	363 (61.7)	128 (54.0)			
MV (%)	No	199 (24.1)	157 (26.7)	42 (17.7)	0.008		0.217
	Yes	626 (75.9)	431 (73.3)	195 (82.3)			
CRRT (%)	No	809 (98.1)	586 (99.7)	223 (94.1)	<0.001		0.324
	Yes	16 (1.9)	2 (0.3)	14 (5.9)			
AKI (%)	No	232 (28.1)	183 (31.1)	49 (20.7)	0.003		0.24
	Yes	593 (71.9)	405 (68.9)	188 (79.3)			
Sepsis (%)	No	417 (50.5)	340 (57.8)	77 (32.5)	<0.001		0.526
	Yes	408 (49.5)	248 (42.2)	160 (67.5)			
VE (%)	No	701 (85.0)	485 (82.5)	216 (91.1)	0.002		0.258
	Yes	124 (15.0)	103 (17.5)	21 (8.9)			
VO (%)	No	790 (95.8)	559 (95.1)	231 (97.5)	0.175		0.127
	Yes	35 (4.2)	29 (4.9)	6 (2.5)			

Statistical characteristics of the validation set							
	Variables	Total (*n* = 290)	0 (*n* = 65)	1 (*n* = 225)	*p*	Test	SMD
Age [mean (SD]		53.27 ± 11.42	51.57 ± 11.42	53.76 ± 11.40	0.174		0.192
LOS [mean (SD]		21.92 ± 23.00	35.88 ± 22.83	17.88 ± 21.46	<0.001		0.812
RR [mean (SD]		19.06 ± 6.81	18.15 ± 5.47	19.32 ± 7.13	0.222		0.184
Rbc [mean (SD]		5.26 ± 9.10	4.67 ± 0.78	5.43 ± 10.32	0.557		0.103
PLT [mean (SD]		179.69 ± 67.95	174.24 ± 67.61	181.26 ± 68.11	0.464		0.104
K [mean (SD]		3.69 ± 0.54	3.73 ± 0.54	3.68 ± 0.53	0.487		0.097
BUN [mean (SD]		15.02 ± 10.93	13.23 ± 4.81	15.54 ± 12.09	0.133		0.252
Glu [mean (SD]		154.54 ± 106.00	138.62 ± 46.80	159.14 ± 117.38	0.17		0.23
K [mean (SD]		138.31 ± 5.00	138.05 ± 4.04	138.39 ± 5.25	0.631		0.072
AG [mean (SD]		14.73 ± 8.94	13.74 ± 6.16	15.02 ± 9.59	0.31		0.159
Sepsis (%)	No	288 (99.3)	65 (100.0)	223 (99.1)	1		0.134
	Yes	2 (0.7)	0 (0.0)	2 (0.9)			

LOS, length of stay; ICU LOS, intensive care unit length of stay; Age, age; Gender, male or female; RBC, red blood cell count; Cr, creatinine; Cl, chloride; PLT, platelet; WBC, white blood cell count; K, potassium; Na, sodium; AG, anion gap; HCO_3_, bicarbonate; BUN, blood urea nitrogen; Glu, blood glucose; HR, heart rate; SBP, systolic blood pressure; DBP, diastolic blood pressure; MAP, mean arterial pressure; RR, respiratory rate; T, temperature; SpO_2_, blood oxygen saturation; GCS, glasgow coma scale; HBP, hypertension; HF, heart failure; RF, renal failure; PVD, peripheral vascular disease; CPD, chronic pulmonary disease; DM, diabetes mellitus; CVD, cerebrovascular disease; NM, nimodipine ICU used; LEV, levetiracetam Icu used; MV, mechanical ventilation; CRRT, continuous renal replacement therapy; AKI, acute kidney injury; Sepsis, sepsis; VE, vascular embolization; VO, vascular occlusion.

**Table 7:** The variable information of the training set and the validation set is included in the model.

Variable	Category/cut-off	Training set		Validation set	
		HR (95% CI)	*P*-value	HR (95% CI)	*P*-value
Sepsis	Present vs. Absent	3.07 (2.21–4.26)	<0.001	2.25 (1.01–5.00)	0.047
Age	≥77.58	2.70 (1.87–3.90)	<0.001	0.90 (0.28–2.89)	0.862
	56.69–77.58	1.36 (0.96–1.93)	0.085	1.01 (0.75–1.37)	0.947
AG	≥18.00	2.26 (1.30–3.93)	0.004	1.05 (0.73–1.51)	0.81
	12.00–18.00	1.06 (0.68–1.65)	0.792	0.97 (0.70–1.34)	0.832
Glu	≥218.00	2.05 (1.35–3.12)	0.001	0.78 (0.48–1.27)	0.309
	118.00–218.00	1.81 (1.31–2.50)	<0.001	0.96 (0.70–1.33)	0.821
RR	≥22.00	1.99 (1.32–2.99)	0.001	1.71 (1.15–2.54)	0.008
	15.50–22.00	1.50 (1.08–2.09)	0.016	1.02 (0.72–1.46)	0.892
BUN	≥24.00	1.61 (1.08–2.39)	0.019	1.78 (1.11–2.86)	0.017
	17.00–24.00	1.50 (1.08–2.08)	0.016	1.51 (1.04–2.20)	0.03
Rbc	≥3.96	0.47 (0.32–0.67)	<0.001	2.07 (0.88–4.84)	0.094
	3.37–3.96	0.57 (0.39–0.84)	0.004	2.15 (0.84–5.51)	0.11
LOS	≥15.09	0.05 (0.03–0.08)	<0.001	<0.01 (<0.01–0.03)	0.022
	3.96–15.09	0.09 (0.06–0.13)	<0.001	<0.01 (<0.01–0.14)	0.059
PLT	≥186.00	0.63 (0.43–0.93)	0.02	1.08 (0.77–1.52)	0.645
	137.00–186.00	0.73 (0.48–1.10)	0.135	1.02 (0.69–1.50)	0.924
K	3.50–4.50	0.61 (0.43–0.86)	0.005	0.60 (0.28–1.28)	0.182
	≥4.50	0.80 (0.51–1.27)	0.354	0.82 (0.60–1.11)	0.19
Na	≥141.00	1.19 (0.82–1.72)	0.352	1.19 (0.82–1.73)	0.352
	137.00–141.00	0.69 (0.49–0.96)	0.03	0.69 (0.49–0.96)	0.03

LOS, length of stay; Age, age; RBC, red blood cell count; PLT, platelet; K, potassium; Na, sodium; AG, anion gap; BUN, blood urea nitrogen; Glu, blood glucose; RR, respiratory rate; Sepsis, sepsis.

### Construction and validation of the nomogram

**Figure 4:**
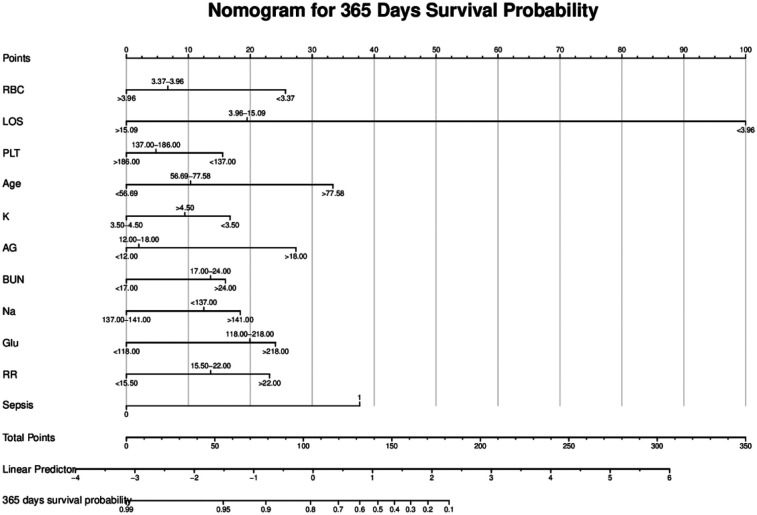
Nomogram model. RBC, red blood cell count; LOS, length of stay; PLT, platelet; Age, age; K, potassium; AG, anion gap; BUN, blood urea nitrogen; Na, sodium; Glu, blood glucose; RR, respiratory rate; Sepsis, sepsis.

As illustrated in Figure 4, the nomogram demonstrates a robust capability to predict the one-year survival prognosis of patients with non-traumatic subarachnoid hemorrhage. This model integrates multiple prognostic factors, with hospital stay duration exerting the most significant influence on prognosis, followed by sepsis, age, blood glucose levels, respiratory rate, anion gap, red blood cell count, sodium and potassium concentrations, urea nitrogen, and platelet count. The cumulative score derived from these independent prognostic factors is positively correlated with the one-year mortality rate.

**Figure 5:**
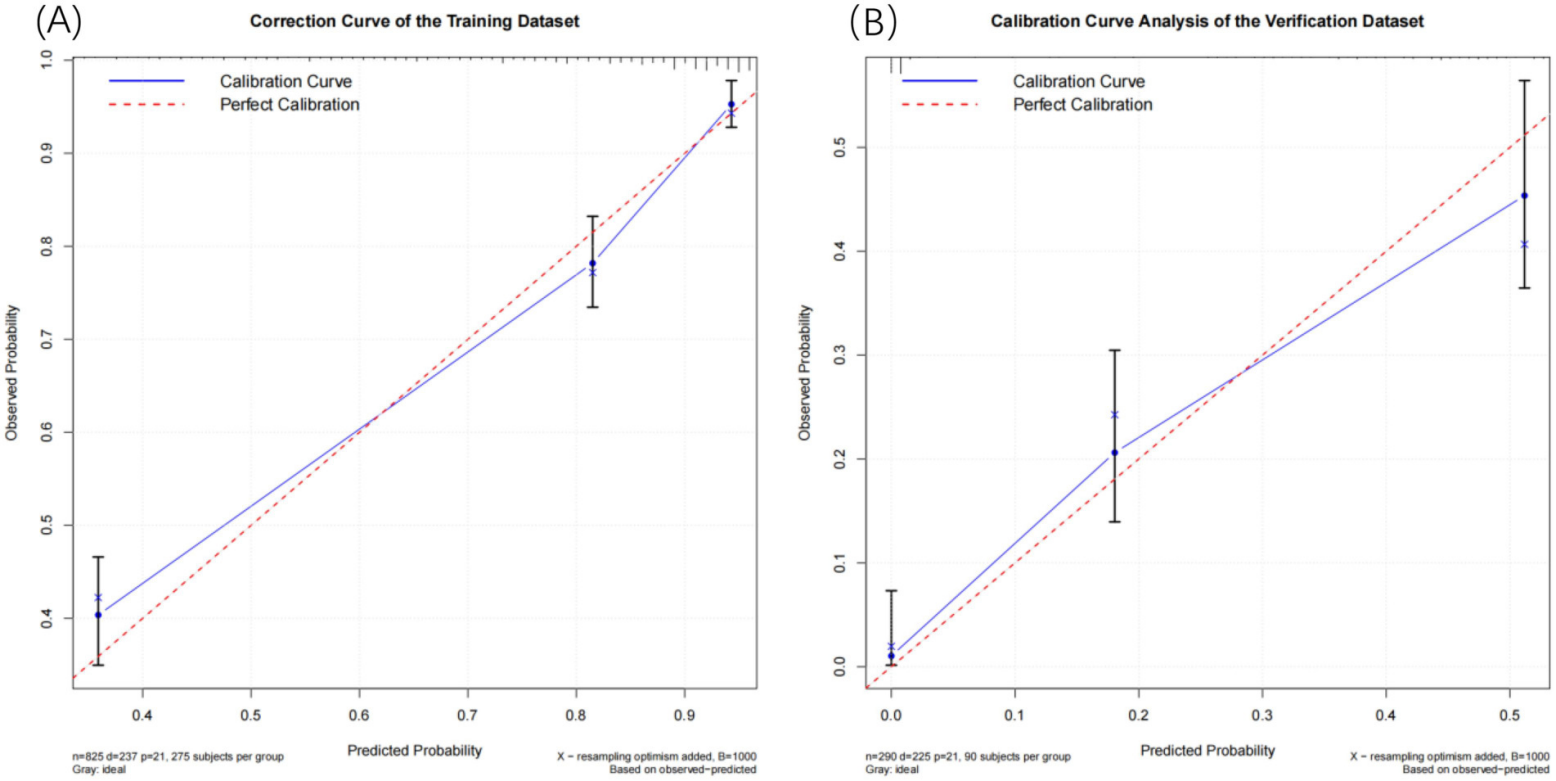
**(A)** Training set calibration curve and **(B)** validation set calibration curve.

To minimize data bias and error, we employed the Bootstrap resampling method to generate calibration curves for both the training set and the validation set (Figure 5). We conducted 1,000 iterations to mitigate overfitting risks. The calibration plots indicate that the 365-day survival prediction model closely aligns with observed outcomes. A thorough analysis confirms that this predictive model exhibits excellent calibration accuracy.

**Figure 6:**
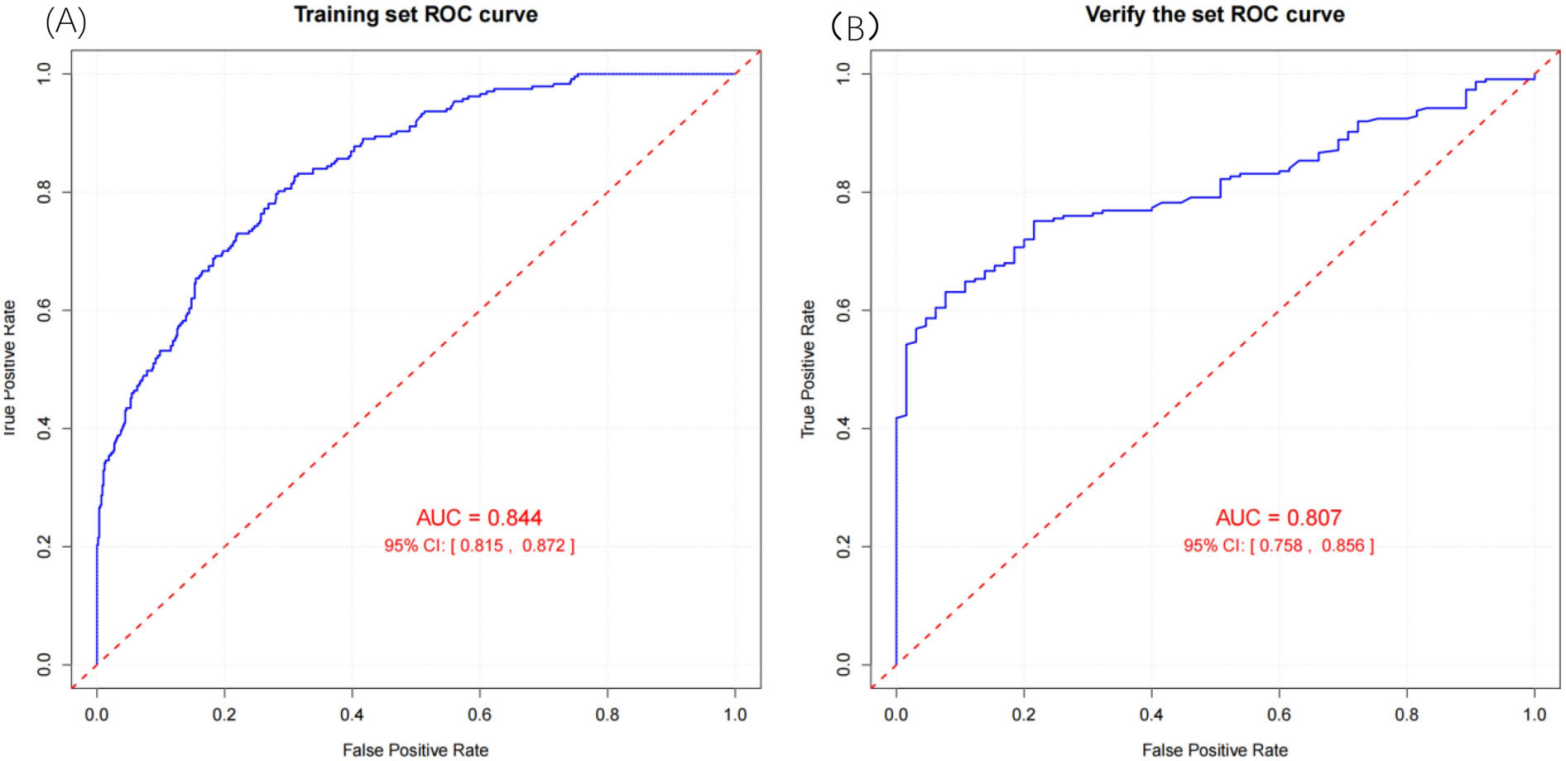
**(A)** Validation set ROC curve and **(B)** training set ROC curve.

**Figure 7:**
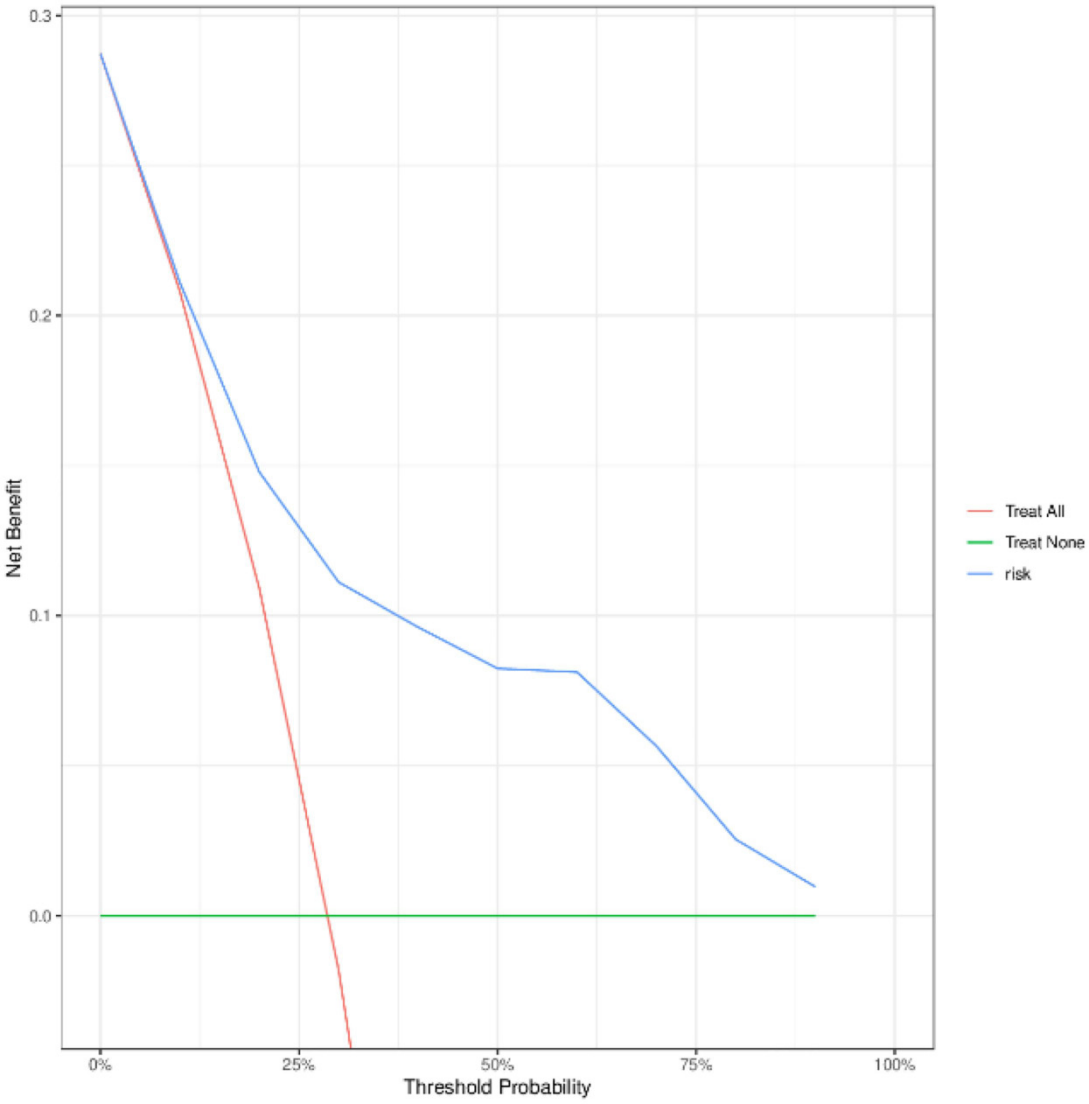
Training set DCA curve.

**Figure 8:**
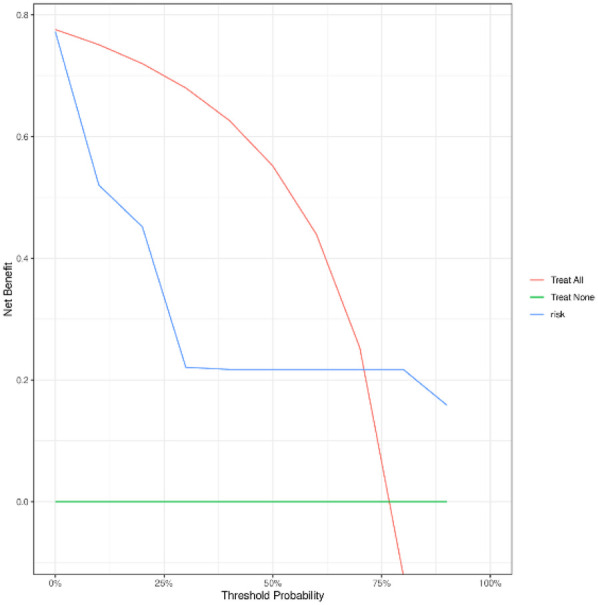
Validation set DCA curve.

To evaluate the performance of the nomogram model, we conducted receiver operating characteristic (ROC) curve analysis. As shown in Figure 6, the area under the curve (AUC) for the training set was 0.844 (95% CI: 0.815–0.872), with a C-index of 0.827 (95% CI: 0.803–0.851). These metrics suggest that the model exhibits satisfactory discriminatory power. To further validate the model's performance, we applied it to an external validation set. As illustrated in Figure 8, the AUC was 0.807 (95% CI: 0.758–0.856), and the C-index was 0.851 (95% CI: 0.825–0.875), confirming its robust discriminatory capability. Additionally, decision curve analysis (DCA) was performed to assess the clinical utility of the nomogram. The DCA results for both the training set (Figure 7) and the external validation set (Figure 8) demonstrate that the risk model provides significant net benefits within a specific threshold range.

**Figure 9:**
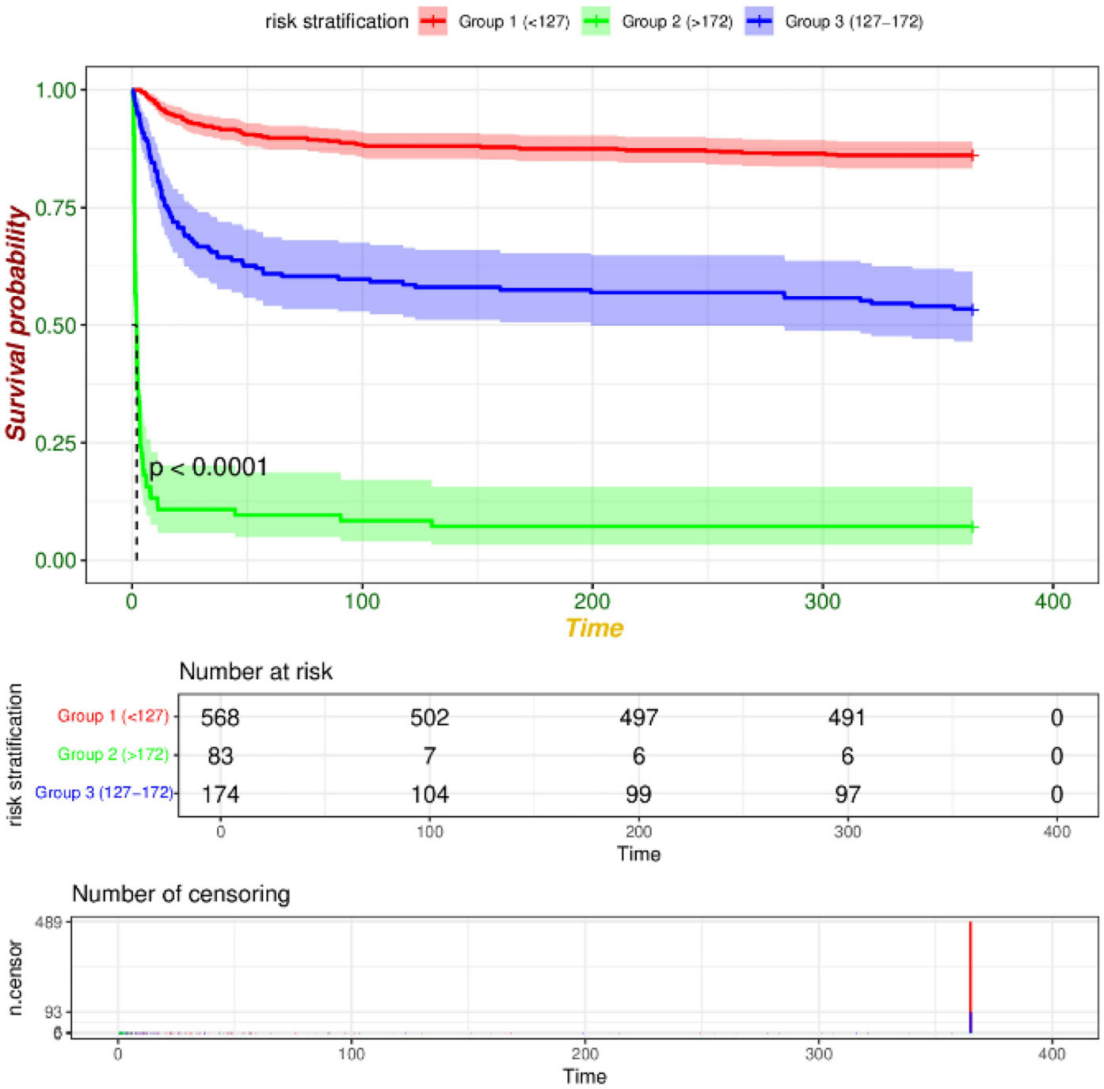
KM curve of training set risk stratification.

**Figure 10:**
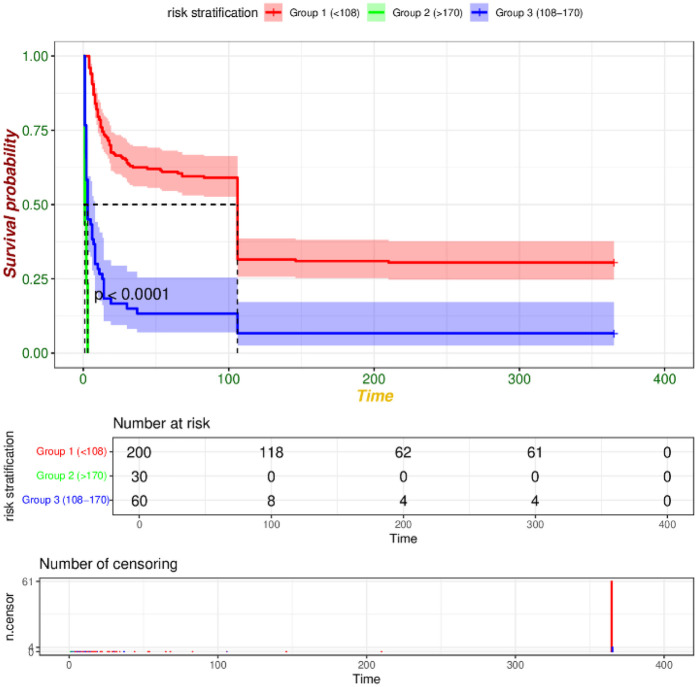
Curve of validation set risk stratification.

We utilized R software and RStudio to compute the score for each risk factor, subsequently aggregating these scores to derive the total index. The X-tile software was then employed to determine the optimal cut-off value for risk stratification. In the training set, patients were categorized into three risk groups based on their 365-day survival prediction model: low-risk (<127 points), medium-risk (127–172 points), and high-risk (>172 points). In the validation set, the corresponding categories were defined as low-risk (<108 points), medium-risk (108–170 points), and high-risk (>170 points). Kaplan–Meier survival curve analyses of the risk stratifications in both the training set (Figure 9) and the validation set (Figure 10) revealed that the 365-day survival probability of the low-risk group was significantly higher compared to the other two groups, with statistically significant differences (*P* < 0.001).

## Discussion

The main novelty of this study lies in the development and external validation of an easy-to-use nomogram using Cox regression, based on a large-scale, international ICU database. This model integrates patients' baseline characteristics, laboratory findings, and other clinical indicators. It not only confirms established prognostic factors such as age, but more importantly, systematically evaluates and externally validates the prognostic value of routinely collected clinical parameters, ultimately providing a validated and visually accessible clinical tool.

A study by Bergamini Carlo et al. reported that approximately 22% of subarachnoid hemorrhage (SAH) patients died within one year post-diagnosis (19). In our study, the internal cohort comprised 825 non-traumatic SAH patients from the MIMIC-IV database, with a one-year mortality rate of 28.7%—notably higher than previously reported rates. This discrepancy may be attributed to factors such as the exclusion of incomplete cases during data processing, the extended timeframe of the database, and rapid advancements in medical technology that could have influenced outcomes. We analyzed baseline and clinical data from non-traumatic SAH patients in MIMIC-IV to identify independent survival risk factors and subsequently developed a nomogram model. External validation was performed using data from non-traumatic SAH patients admitted to Qinghai Provincial People's Hospital (China) over the past decade. Results identified the following independent risk factors for 365-day survival: hospital stay duration, age, respiratory rate, red blood cell count, platelet count, potassium, sodium, anion gap, blood urea nitrogen, glucose levels, and sepsis. External validation confirmed the model's discrimination and calibration. Despite potential differences between internal/external datasets (e.g., racial variations, admission/discharge timing, follow-up information), the model demonstrated favorable accuracy and broad applicability.

Hospitalization duration is a critical indicator of healthcare quality. An inappropriately short stay may compromise treatment efficacy, whereas an unnecessarily prolonged stay substantially increases complication risks, representing an inherent contradiction in medical practice. Ali Alaraj et al. reported that subarachnoid hemorrhage (SAH) patients often require hospitalization exceeding three weeks for adequate clinical vasospasm monitoring (20). Accurate early prediction of hospitalization duration and outcomes is essential for optimizing clinical management and resource allocation. Bambang Tri Prasetyo et al. addressed this by developing a scoring system evaluating four factors: high-grade aSAH, aneurysm treatment methods, cardiovascular comorbidities, and hospital-acquired pneumonia in SAH patients (21). This system improves hospitalization length prediction accuracy, enabling timely treatment adjustments, better outcomes, and potential cost reduction. Our study found hospital stays <3.96 days significantly reduced survival probability. Conversely, stays >15.09 days (HR = 0.0509, *p* < 0.0001) and 3.96–15.09 days (HR = 0.0909, *p* < 0.0001) correlated with lower adverse outcome incidence. Prognosis varied by age and sex: Charlotte H. Harrison et al. reported higher SAH risk in younger males vs. females, but reversed in patients >75 years (22). Yuan Yuan et al. found significantly higher mean age in poor-prognosis SAH patients (23). Our results indicate SAH patients >77.58 years (HR = 2.700, *p* < 0.0001) have substantially increased adverse outcome risks, potentially due to age-related organ degeneration and physiological decline. Therefore, treatment plans should be age-adapted with sufficient monitoring to prevent complications.

Rapid breathing is a significant risk factor for lung injury and acute respiratory distress syndrome (ARDS) (24). Hyperventilation, a frequent complication in subarachnoid hemorrhage (SAH) patients, is significantly correlated with delayed cerebral ischemia and poor prognosis after discharge (25). Specifically, respiratory rates >22.00 breaths per minute (HR = 1.9912, *p* = 0.0009) and 15.50–22.00 breaths per minute (HR = 1.4997, *p* = 0.0161) are associated with progressively worse prognoses. Wenyuan Du et al. demonstrated a significant association between hypothermia and in-hospital mortality (26), while other studies recommend maintaining body temperature between 36.0 °C and 37.5 °C (27, 28). Respiratory rate is thus a critical risk factor for survival prognosis in non-traumatic SAH, whereas the impact of body temperature remains inconclusive. In ICU management, ensuring stable admission vital signs is crucial for optimal outcomes.

Jiuling Liu et al. demonstrated that decreased hemoglobin to red cell distribution width ratio (HRR) significantly correlates with elevated mortality risk in non-traumatic SAH patients (29). Long Zhao et al. emphasized that reduced red blood cell (RBC) count increases delayed cerebral ischemia (DCI) risk (30). While Longyuan Gu et al. identified low RBC count as a significant factor for higher 7-day mortality in SAH (31). Wanwan Zhang et al. reported that elevated admission white blood cell-to-platelet ratio associates with unfavorable prognosis (32). Bappaditya Ray et al. further established that platelet elevation magnitude correlates with adverse clinical outcomes (33). Microthrombosis post-aneurysmal SAH (aSAH) is a recognized mechanism contributing to DCI development (33), with elevated platelet indices linked to increased DCI risk. Existing literature suggests antiplatelet therapy may mitigate this risk (30). In our study, RBC and platelet counts were integrated into an aSAH adverse outcome prediction model. Specifically, adverse outcome risk significantly increased when RBC count was <3.37 × 10^12^/L and platelet count <137 × 10^9^/L. Conversely, risk decreased when RBC count was 3.37–3.96 × 10^12^/L (HR = 0.5680, *P* = 0.0041), >3.96 × 10^12^/L (HR = 0.4654, *P* < 0.0001), or platelet count >186 × 10^9^/L (HR = 0.6294, *P* = 0.0196). Markedly low platelet counts may impair coagulation and increase rebleeding risk. The relationship between RBCs, platelets, and survival prognosis in aSAH patients remains inconclusive, warranting further prospective trials to validate findings and guide clinical practice.

Patients with subarachnoid hemorrhage (SAH) commonly develop electrolyte imbalances. Stress responses frequently induce hypokalemia, activating the Na⁺/K⁺-ATPase pump and causing intracellular potassium sequestration that exacerbates the condition (34). This process may detrimentally affect cerebrovascular cells, increasing rebleeding risk (35). Studies using the MIMIC database by Haoxin Liu et al. identified the admission blood urea nitrogen-to-potassium (BUN/K) ratio as a significant predictor of 30-day all-cause mortality in non-traumatic SAH (36). Hyun Min Jung et al. demonstrated that the admission glucose-to-potassium ratio (GPR) predicts 3-month mortality in aneurysmal SAH (aSAH) (37). Dongcai Jin et al. reported a significant correlation between admission serum sodium levels and in-hospital mortality (38), while Wenyuan Du et al. found baseline bicarbonate levels negatively correlated with 30-day mortality in non-traumatic SAH (39). Changli Zhong et al. discovered that elevated anion gap (AG) levels at ICU admission significantly associate with increased all-cause mortality in non-traumatic SAH (40). Our study shows potassium concentrations of 3.5–4.5 mmol/L (HR = 0.6074, *p* = 0.0054) and sodium concentrations of 137–141 mmol/L (HR = 0.6892, *p* = 0.0298) correlate with reduced adverse outcome risk. Conversely, anion gap >18 mmol/L (HR = 2.26, *p* = 0.0039) associates with significantly increased risk. These findings underscore the critical role of electrolyte homeostasis in clinical management.

Blood urea nitrogen (BUN), a protein metabolism byproduct, reflects overall metabolic status and renal function. Elevated BUN levels after subarachnoid hemorrhage (SAH) may indicate renal impairment or other physiological disturbances (36). Yuan et al. demonstrated significantly higher urea levels in patients with poor prognoses vs. favorable outcomes (23). Specifically, compared to lower levels, mortality significantly increased when BUN was 17–24 mmol/L (HR = 1.4985, *P* = 0.0161) or >24 mmol/L (HR = 1.6093, *P* = 0.0190), indicating a positive correlation between elevated BUN and mortality risk in SAH. Thus, serum BUN serves as a reliable predictor of survival prognosis in SAH patients, highlighting the importance of comprehensive renal evaluation. Further prospective large-scale studies are warranted to validate the relationship between BUN levels and survival outcomes.

Previous research has established that hyperglycemia following subarachnoid hemorrhage (SAH) can induce secondary brain injury and cerebral vasospasm. Studies have demonstrated an association between post-SAH hyperglycemia and a higher incidence of adverse outcomes, as well as increased mortality. Notably, Zeyu Zhang et al. emphasized that stress-induced hyperglycemia following non-traumatic SAH is significantly correlated with poor prognosis (41) and Chiara Robba et al. reported its association with poorer prognoses (28). Our analysis revealed that in patients with SAH, the risk of adverse outcomes increased significantly when blood glucose levels were within the range of 118.00–218.00 mg/dl (HR = 1.8109, *P* = 0.0003) and when levels exceeded 218.00 mg/dl (HR = 2.0488, *P* = 0.0008). Therefore, close monitoring of blood glucose levels and prompt implementation of interventions for hyperglycemia are imperative in the clinical management of these patients.

Sepsis and delayed cerebral ischemia (DCI) represent significant complications in patients with subarachnoid hemorrhage (SAH). Franz-Simon Centner et al. demonstrated a strong correlation between sepsis, DCI, and functional outcomes in patients with aneurysmal SAH (aSAH), highlighting complex interactions among these conditions that collectively exacerbate adverse outcomes (42). Additionally, Bruno Gonçalves et al. prospectively examined the incidence of sepsis within the first 14 days of hospitalization, reporting a rate of 28%. Their findings indicated a threefold higher risk of adverse outcomes and mortality in patients with sepsis compared to those without, underscoring the pivotal role of sepsis in determining SAH patient prognosis (43). Our analysis revealed that SAH patients with concurrent sepsis (HR = 3.0707, *P* < 0.0001) had a significantly higher risk of adverse outcomes. Consequently, sepsis can be considered a robust predictor of survival prognosis in SAH patients. However, it should be noted that due to data limitations, DCI was not included as a variable in this study. Therefore, further prospective studies with larger sample sizes are warranted to elucidate the complex relationships among sepsis, DCI, and other serious complications.

The core clinical value of this nomogram is to provide physicians with an objective, quantitative tool at the critical decision point of patient discharge, to identify nSAH patients with a high risk of mortality within one year after discharge. It thereby facilitates personalized risk assessment and supports actionable changes in post-acute management. For clinicians, the tool offers an evidence-based basis for formulating individualized and intensified long-term management strategies. High-risk patients may be prioritized for more vigilant care, which could include scheduling earlier and more frequent outpatient reviews, initiating proactive telephone follow-ups to monitor recovery status and medication adherence, as well as facilitating referrals to specialized rehabilitation programs. For patients and their families, being informed of the high-risk status may help reframe the discharge process as the beginning of a structured long-term recovery plan rather than merely the end of acute care. This awareness could foster a deeper understanding of the potential long-term seriousness of the disease, thereby potentially enhancing motivation to adhere to medical recommendations. Day-to-day support such as assisting with medication compliance, participating in rehabilitation exercises, and maintaining a health-promoting lifestyle can enable families to provide more targeted care. From a health-system perspective, the risk stratification enabled by the nomogram may help guide the allocation of more intensive support and resources to higher-risk patients, potentially improving the effectiveness of post-discharge care. Although this nomogram has undergone external validation, demonstrating its reliability and generalizability, the actual effect of these proposed behavioral changes on long-term patient outcomes requires further confirmation through prospective clinical studies.

Although the model demonstrated robust performance in external validation, its predictions in different healthcare settings must be interpreted with caution. In the United States, ICU systems implemented multimodal monitoring and standardized vasospasm prevention protocols earlier (6, 44). Conversely, in China, particularly in the Qinghai region, ICUs are often constrained by resources and may rely more heavily on clinical assessment and intermittent monitoring. Furthermore, while medical centers in the United States typically have dedicated neurointerventional teams, patients in the Qinghai region experience longer transfer times to facilities with interventional capabilities. Additionally, family members of Chinese patients often actively advocate for continued treatment during decision-making. While this may prolong patient survival, it is frequently accompanied by a decline in functional prognosis (45). These systemic differences in healthcare, coupled with the aforementioned data limitations, suggest that the model may perform better in high-resource settings than in resource-constrained regions. Collectively, these factors define the boundary conditions for model application. Future multi-center prospective studies are needed to further optimize the model.

This study developed and validated a nomogram model to predict the one-year survival prognosis of patients with non-traumatic subarachnoid hemorrhage. The model demonstrated good predictive performance and was effective. However, several limitations should be acknowledged. First, the retrospective design inevitably introduced uncontrollable confounding factors, leading to potential selection bias. The validation cohort was derived from a single center and had a relatively small sample size (*n* = 290). Consequently, the model's performance requires further validation across diverse populations, including patients of different races, regions, and varying levels of healthcare resources. Second, key clinical scores were unavailable, including Hunt-Hess grade, modified Fisher scale, admission modified Rankin Scale (mRS) score, and detailed imaging data. This absence may impact the model in several ways: (a) inaccurate risk prediction for patients whose clinical manifestations diverge from laboratory indicators; (b) inability to identify the elevated risk of delayed cerebral ischemia specifically in patients with Fisher grade 3–4; and (c) potential underestimation of the non-linear impact of consciousness level on prognosis due to the treatment of the Glasgow Coma Scale (GCS) score as a binary variable. Finally, adverse outcomes in this study were defined solely as mortality, excluding functional prognosis measures (e.g., mRS) or quality-of-life indicators. This narrow definition may result in insufficient assessment of the clinical value for patients who survive with severe disability. Collectively, these factors limit the generalizability of our findings and may introduce bias. Future large-scale prospective multicenter studies incorporating imaging scores and functional prognosis indicators are warranted to overcome these limitations and further refine the model.

## Conclusion

We developed a nomogram model utilizing the MIMIC-IV database to predict the survival prognosis of patients with non-traumatic subarachnoid hemorrhage (SAH) and validated it externally using data from Qinghai Provincial People's Hospital. The nomogram exhibited robust predictive performance in both internal and external validation cohorts. Identified independent risk factors encompassed length of hospital stay, age, respiratory rate, red blood cell count, platelet count, potassium levels, sodium levels, anion gap, urea nitrogen, blood glucose levels, and the presence of sepsis. This model can assist clinicians in evaluating patient prognosis, serve as a valuable reference for clinical decision-making, and facilitate individualized risk assessment.

## Data Availability

Publicly available datasets were analyzed in this study. This data can be found here: https://physionet.org/
