# Supplementary figures and images for: Development and external validation of a nomogram for predicting one-year survival in patients with non-traumatic subarachnoid hemorrhage
Source: Front Surg. 2025 Sep 12;12:1579429. doi: 10.3389/fsurg.2025.1579429 (PMC12477427; doi:10.3389/fsurg.2025.1579429)

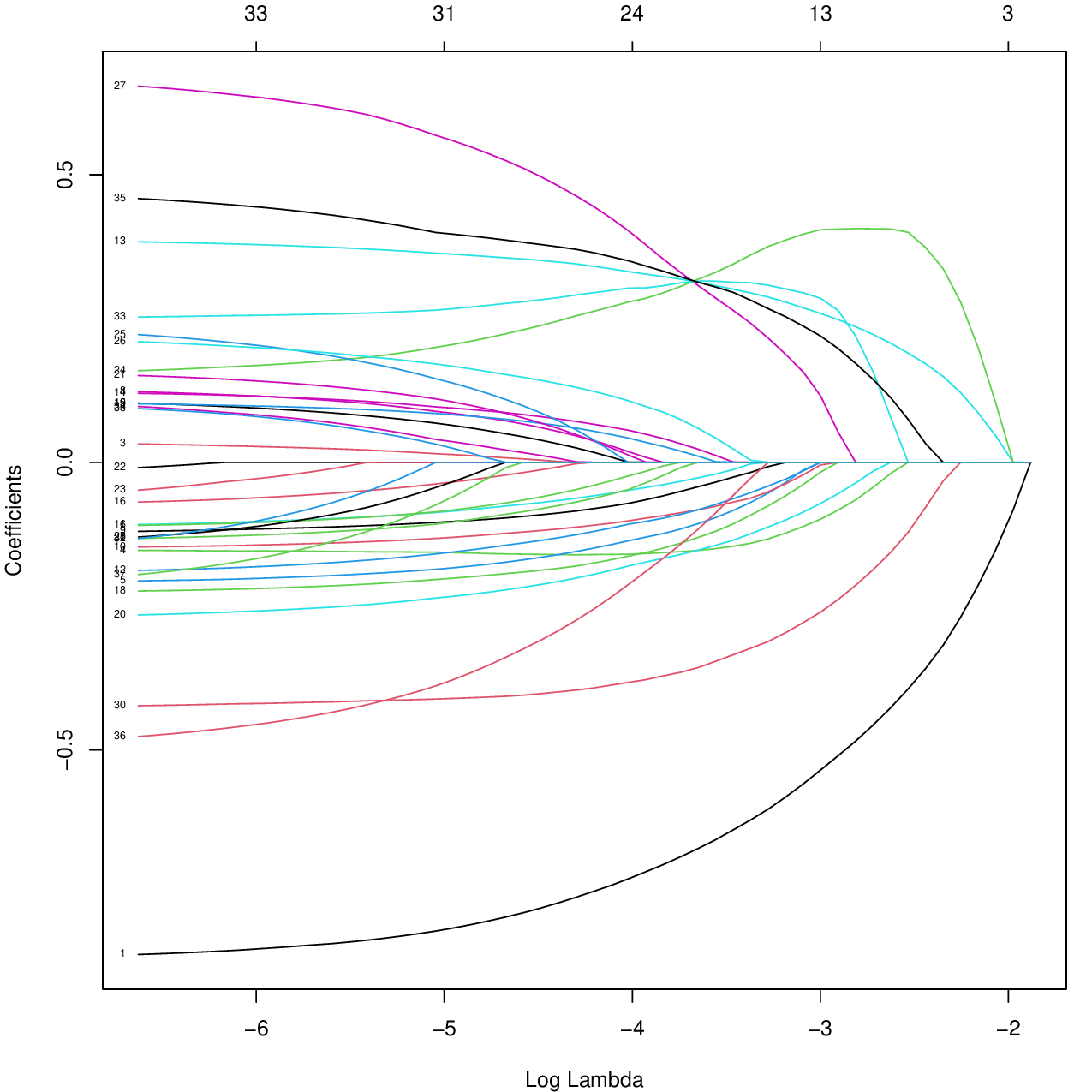

Supplement: Supplementary file 1 [file Image1.tiff]

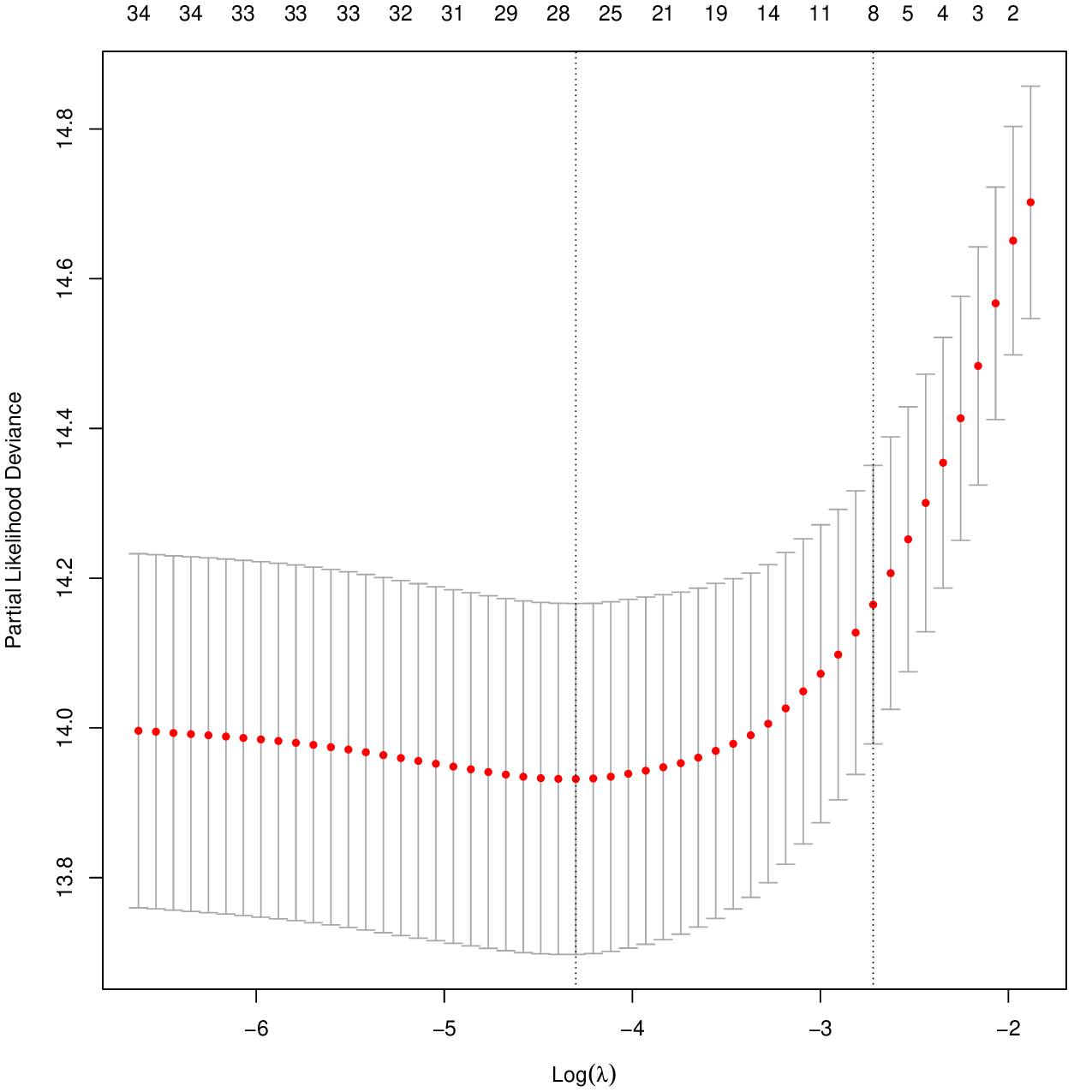

Supplement: Supplementary file 2 [file Image2.tiff]
