# Supplementary material for: Development and external validation of a nomogram for predicting one-year survival in patients with non-traumatic subarachnoid hemorrhage
Source: Front Surg. 2025 Sep 12;12:1579429. doi: 10.3389/fsurg.2025.1579429 (PMC12477427; doi:10.3389/fsurg.2025.1579429)
Supplement: Supplementary file 3 [file Table1.docx]

| Variables | Beta | SE | Z_value | P_value | HR | CI_lower | CI_upper |
| --- | --- | --- | --- | --- | --- | --- | --- |
| LOS >15.09 | -2.9750 | 0.2819 | -10.5549 | 0.0001 | 0.0510 | 1.0298 | 1.0927 |
| LOS 3.96-15.09 | -2.2817 | 0.2294 | -9.9470 | 0.0000 | 0.1021 | 1.0673 | 1.1736 |
| ICU LOS >4.88 | -0.2982 | 0.2666 | -1.1185 | 0.2634 | 0.7422 | 1.5530 | 3.4954 |
| ICU LOS 2.04-4.88 | -0.3485 | 0.2299 | -1.5161 | 0.1295 | 0.7057 | 1.5679 | 3.0266 |
| Age >77.58 | 0.8924 | 0.2055 | 4.3424 | 0.0001 | 2.4411 | 5.1126 | 38.5455 |
| Age 56.69-77.58 | 0.2577 | 0.1834 | 1.4054 | 0.1599 | 1.2940 | 2.4677 | 6.3832 |
| RBC >3.96 | -0.7773 | 0.2035 | -3.8197 | 0.0001 | 0.4596 | 1.3613 | 1.9836 |
| RBC 3.37-3.96 | -0.5594 | 0.2111 | -2.6493 | 0.0081 | 0.5716 | 1.4592 | 2.3739 |
| Cr >1.10 | 0.0731 | 0.3427 | 0.2133 | 0.8311 | 1.0758 | 1.7326 | 8.2139 |
| Cr 0.60-1.10 | -0.1918 | 0.2640 | -0.7266 | 0.4675 | 0.8255 | 1.6356 | 3.9945 |
| Cl >106.00 | -0.3031 | 0.2406 | -1.2599 | 0.2077 | 0.7385 | 1.5854 | 3.2656 |
| Cl 103.00-106.00 | -0.2004 | 0.1976 | -1.0145 | 0.3104 | 0.8184 | 1.7431 | 3.3380 |
| PLT >186.00 | -0.4869 | 0.2219 | -2.1945 | 0.0282 | 0.6145 | 1.4886 | 2.5839 |
| PLT 137.00-186.00 | -0.2758 | 0.2368 | -1.1647 | 0.2441 | 0.7590 | 1.6116 | 3.3440 |
| K >4.50 | -0.0479 | 0.2547 | -0.1882 | 0.8507 | 0.9532 | 1.7836 | 4.8077 |
| K 3.50-4.50 | -0.3472 | 0.1891 | -1.8362 | 0.0663 | 0.7067 | 1.6288 | 2.7834 |
| Na >141.00 | 0.4372 | 0.2600 | 1.6813 | 0.0927 | 1.5483 | 2.5348 | 13.1626 |
| Na 137.00-141.00 | -0.2088 | 0.2002 | -1.0428 | 0.2970 | 0.8116 | 1.7301 | 3.3254 |
| AG >18.00 | 0.4591 | 0.3511 | 1.3076 | 0.1910 | 1.5826 | 2.2151 | 23.3167 |
| AG 12.00-18.00 | -0.0534 | 0.2434 | -0.2195 | 0.8263 | 0.9480 | 1.8010 | 4.6066 |
| HCO₃ >22.00 | -0.3076 | 0.2436 | -1.2630 | 0.2066 | 0.7352 | 1.5779 | 3.2708 |
| HCO₃ 20.00-22.00 | -0.1437 | 0.2309 | -0.6224 | 0.5337 | 0.8662 | 1.7348 | 3.9033 |
| BUN >24.00 | 0.4766 | 0.2736 | 1.7418 | 0.0815 | 1.6105 | 2.5652 | 15.6964 |
| BUN 17.00-24.00 | 0.3888 | 0.1787 | 2.1763 | 0.0295 | 1.4753 | 2.8275 | 8.1165 |
| Glu >218.00 | 0.6516 | 0.2492 | 2.6150 | 0.0089 | 1.9186 | 3.2456 | 22.7996 |
| Glu 118.00-218.00 | 0.4558 | 0.1802 | 2.5295 | 0.0114 | 1.5774 | 3.0285 | 9.4463 |
| HR >98.00 | 0.2532 | 0.2196 | 1.1529 | 0.2489 | 1.2882 | 2.3107 | 7.2510 |
| HR 76.00-98.00 | 0.1967 | 0.1689 | 1.1642 | 0.2443 | 1.2173 | 2.3970 | 5.4470 |
| SBP >129.00 | -0.0379 | 0.2348 | -0.1615 | 0.8717 | 0.9628 | 1.8362 | 4.5969 |
| SBP 118.00-129.00 | -0.2203 | 0.2558 | -0.8612 | 0.3891 | 0.8023 | 1.6257 | 3.7605 |
| DBP >65.00 | -0.0034 | 0.2837 | -0.0119 | 0.9905 | 0.9966 | 1.7710 | 5.6848 |
| DBP 57.00-65.00 | 0.0893 | 0.2813 | 0.3174 | 0.7509 | 1.0934 | 1.8776 | 6.6709 |
| MAP >80.00 | 0.1001 | 0.3455 | 0.2896 | 0.7721 | 1.1052 | 1.7534 | 8.8056 |
| MAP 70.50-80.00 | -0.3620 | 0.3244 | -1.1159 | 0.2645 | 0.6963 | 1.4458 | 3.7247 |
| RR >22.00 | 0.6014 | 0.2303 | 2.6109 | 0.0090 | 1.8246 | 3.1955 | 17.5595 |
| RR 15.50-22.00 | 0.3670 | 0.1800 | 2.0389 | 0.0415 | 1.4434 | 2.7574 | 7.7991 |
| T >37.44 | -0.4901 | 0.2372 | -2.0657 | 0.0389 | 0.6126 | 1.4693 | 2.6517 |
| T 36.61-37.44 | -0.3325 | 0.1568 | -2.1209 | 0.0339 | 0.7171 | 1.6945 | 2.6514 |
| HF | -0.3212 | 0.2416 | -1.3295 | 0.1837 | 0.7253 | 1.5711 | 3.2043 |
| RF | -0.1545 | 0.2315 | -0.6674 | 0.5045 | 0.8568 | 1.7233 | 3.8532 |
| CPD | 0.2532 | 0.1866 | 1.3574 | 0.1747 | 1.2882 | 2.4441 | 6.4039 |
| DM | -0.1531 | 0.2048 | -0.7476 | 0.4547 | 0.8580 | 1.7760 | 3.6032 |
| MV | 0.1985 | 0.2007 | 0.9890 | 0.3226 | 1.2196 | 2.2771 | 6.0957 |
| NM | -0.0502 | 0.1648 | -0.3045 | 0.7607 | 0.9511 | 1.9910 | 3.7194 |
| LEV | 0.0005 | 0.1544 | 0.0032 | 0.9974 | 1.0005 | 2.0944 | 3.8731 |
| CRRT | -0.3142 | 0.3785 | -0.8301 | 0.4065 | 0.7304 | 1.4160 | 4.6352 |
| AKI | 0.3000 | 0.1983 | 1.5132 | 0.1302 | 1.3499 | 2.4973 | 7.3233 |
| Sepsis | 1.0895 | 0.1869 | 5.8279 | 0.0001 | 2.9728 | 7.8523 | 72.8507 |
| VE | -0.0858 | 0.2527 | -0.3395 | 0.7342 | 0.9178 | 1.7494 | 4.5088 |

Table 1 Single factor analysis Meaningful factor multiple factor analysis Abbreviations: LOS = Length of Stay;ICU LOS = Intensive Care Unit Length of Stay;Age = Age;RBC = Red Blood Cell count;Cr = Creatinine;Cl = Chloride;PLT= Platelet;K = Potassium;Na = Sodium;AG = Anion Gap;HCO₃= Bicarbonate;BUN = Blood Urea Nitrogen;Glu = Blood Glucose;HR = Heart Rate;SBP = Systolic Blood Pressure;DBP = Diastolic Blood Pressure;MAP = Mean Arterial Pressure;RR = Respiratory Rate;T = Temperature;HF = Heart Failure;RF = Renal Failure;CPD = Chronic Pulmonary DiseaseDM = Diabetes Mellitus;CVD = Cerebrovascular Disease;NM = Nimodipine ICU Used;LEV = Levetiracetam Icu Used;MV = Mechanical Ventilation;CRRT = Continuous Renal Replacement Therapy;AKI = Acute Kidney Injury;Sepsis = Sepsis;VE= Vascular Embolization.
